# Metabolic Profiling of Thymic Epithelial Tumors Hints to a Strong Warburg Effect, Glutaminolysis and Precarious Redox Homeostasis as Potential Therapeutic Targets

**DOI:** 10.3390/cancers14061564

**Published:** 2022-03-18

**Authors:** Mohammad Alwahsh, Robert Knitsch, Rosemarie Marchan, Jörg Lambert, Christian Hoerner, Xiaonan Zhang, Berthold Schalke, De-Hyung Lee, Elena Bulut, Thomas Graeter, German Ott, Katrin S. Kurz, Gerhard Preissler, Sebastian Schölch, Joviana Farhat, Zhihan Yao, Carsten Sticht, Philipp Ströbel, Roland Hergenröder, Alexander Marx, Djeda Belharazem

**Affiliations:** 1 Leibniz-Institut für Analytische Wissenschaften—ISAS-e.V., 44139 Dortmund, Germany; r.knitsch@gmx.de (R.K.); jlambert@lambertnet.de (J.L.); roland.hergenroeder@isas.de (R.H.); 2 Institute of Pathology and Medical Research Center (ZMF), University Medical Center Mannheim, Heidelberg University, 68167 Mannheim, Germany; christian.hoerner@umm.de (C.H.); dr_zhangxn@outlook.com (X.Z.); zhihan.yao@medma.uni-heidelberg.de (Z.Y.); alexander.marx@umm.de (A.M.); 3 Department of Pharmacy, Faculty of Pharmacy, Al-Zaytoonah University of Jordan, P.O. Box 130, Amman 11733, Jordan; 4 Department of Toxicology, Leibniz Research Center for Working Environment and Human Factors at the TU Dortmund (IfADo), 44139 Dortmund, Germany; marchan@ifado.de; 5 Department of Neurology, University of Regensburg, 93053 Regensburg, Germany; berthold.schalke@medbo.de (B.S.); de-hyung.lee@medbo.de (D.-H.L.); 6 Department of Thoracic Surgery, Thoraxklinik at Heidelberg University Hospital, 69120 Heidelberg, Germany; elena.bulut@med.uni-heidelberg.de; 7 Thoracic Surgery, Clinic Löwenstein, 74245 Löwenstein, Germany; thomas.graeter@klinik-loewenstein.de; 8 Department of Clinical Pathology, Robert-Bosch-Krankenhaus and Dr. Margarete-Fischer-Bosch Institute of Clinical Pharmacology, 70376 Stuttgart, Germany; german.ott@rbk.de (G.O.); katrin.kurz@rbk.de (K.S.K.); 9 Department of Thoracic Surgery, RBK Lungenzentrum Stuttgart, Robert Bosch Hospital, Clinic Schillerhoehe, 70839 Gerlingen, Germany; gerhard.preissler@rbk.de; 10 DZL, German Center for Lung Research (Deutsches Zentrum fuer Lungenforschung), Department of Thoracic Surgery, Ludwig-Maximilians University Munich, 81377 Munich, Germany; 11 JCCU Translational Surgical Oncology (A430), German Cancer Research Center (DKFZ), 69120 Heidelberg, Germany; sebastian.schoelch@umm.de; 12 DKFZ-Hector Cancer Institute at University Medical Center Mannheim, Heidelberg University, 68167 Mannheim, Germany; 13 Department of Surgery, University Medical Center Mannheim, Medical Faculty Mannheim, Heidelberg University, 68167 Mannheim, Germany; 14 Department of Pharmacy, Faculty of Pharmacy, Al Ain University, Abu Dhabi P.O. Box 122612, United Arab Emirates; joviana.farhat@aau.ac.ae; 15 NGS Core Facility, Medical Faculty Mannheim, Heidelberg University, 68167 Mannheim, Germany; carsten.sticht@medma.uni-heidelberg.de; 16 Institute of Pathology, University Medical Center Göttingen, University of Göttingen, 37075 Göttingen, Germany; philipp.stroebel@med.uni-goettingen.de

**Keywords:** thymoma, thymic carcinoma, HRMAS ^1^H-NMR, metabolomics, biomarker

## Abstract

**Simple Summary:**

Thymomas and thymic carcinomas (TCs) are malignant thymic epithelial tumors (TETs) with poor outcome, if non-resectable. Metabolic signatures of TETs have not yet been studied and may offer new therapeutic options. This is the first metabolomics investigation on thymic epithelial tumors employing nuclear magnetic resonance spectroscopy of tissue samples. We could detect and quantify up to 37 metabolites in the major tumor subtypes, including acetylcholine that was not previously detected in other non-endocrine cancers. A metabolite-based cluster analysis distinguished three clinically relevant tumor subgroups, namely indolent and aggressive thymomas, as well as TCs. A metabolite-based metabolic pathway analysis also gave hints to activated metabolic pathways shared between aggressive thymomas and TCs. This finding was largely backed by enrichment of these pathways at the transcriptomic level in a large, publicly available, independent TET dataset. Due to the differential expression of metabolites in thymic epithelial tumors versus normal thymus, pathways related to proline, cysteine, glutathione, lactate and glutamine appear as promising therapeutic targets. From these findings, inhibitors of glutaminolysis and of the downstream TCA cycle are anticipated to be rational therapeutic strategies. If our results can be confirmed in future, sufficiently powered studies, metabolic signatures may contribute to the identification of new therapeutic options for aggressive thymomas and TCs.

**Abstract:**

Thymomas and thymic carcinomas (TC) are malignant thymic epithelial tumors (TETs) with poor outcome, if non-resectable. Metabolic signatures of TETs have not yet been studied and may offer new therapeutic options. Metabolic profiles of snap-frozen thymomas (WHO types A, AB, B1, B2, B3, *n* = 12) and TCs (*n* = 3) were determined by high resolution magic angle spinning 1H nuclear magnetic resonance (HRMAS 1H-NMR) spectroscopy. Metabolite-based prediction of active KEGG metabolic pathways was achieved with MetPA. In relation to metabolite-based metabolic pathways, gene expression signatures of TETs (*n* = 115) were investigated in the public “The Cancer Genome Atlas” (TCGA) dataset using gene set enrichment analysis. Overall, thirty-seven metabolites were quantified in TETs, including acetylcholine that was not previously detected in other non-endocrine cancers. Metabolite-based cluster analysis distinguished clinically indolent (A, AB, B1) and aggressive TETs (B2, B3, TCs). Using MetPA, six KEGG metabolic pathways were predicted to be activated, including proline/arginine, glycolysis and glutathione pathways. The activated pathways as predicted by metabolite-profiling were generally enriched transcriptionally in the independent TCGA dataset. Shared high lactic acid and glutamine levels, together with associated gene expression signatures suggested a strong “Warburg effect”, glutaminolysis and redox homeostasis as potential vulnerabilities that need validation in a large, independent cohort of aggressive TETs. If confirmed, targeting metabolic pathways may eventually prove as adjunct therapeutic options in TETs, since the metabolic features identified here are known to confer resistance to cisplatin-based chemotherapy, kinase inhibitors and immune checkpoint blockers, i.e., currently used therapies for non-resectable TETs.

## 1. Introduction

Thymomas and thymic carcinomas (TC) are rare thymic epithelial tumors (TETs) [1]. Thymomas are biologically and histologically unique tumors that show variable degrees of thymus-like features, are commonly associated with paraneoplastic autoimmune diseases and are classified as WHO type A, AB, B1, B2, B3 (and rare other) thymomas. Although all thymomas are now considered malignant, type A, AB and B1 thymomas generally show low tumor stages, follow a more indolent clinical course and are henceforth called “indolent TETs”, while type B2 and B3 thymomas are generally aggressive cancers. TCs, among which thymic squamous cell carcinoma is the most common subtype, have no thymus-like features, are only rarely associated with autoimmune diseases, and are histologically indistinguishable from comparable carcinomas in other organs. Nevertheless, they show significant differences on the genetic level, suggesting a unique pathogenesis, not only of thymomas but TCs as well [1,2,3,4,5]. So far, targetable mutations have been observed in less than 5% of TETs, leading to unsatisfactory treatment results in non-resectable tumors [1]. Type B2 and B3 thymomas, together with TCs are henceforth called “aggressive TETs”. Re-programming of cellular energy metabolism to support continuous cell growth and proliferation has emerged as a hallmark of many cancers [6,7]. The approach of metabolomics, i.e., the study of a subset of small molecules derived from the global or targeted analysis of metabolic profiles from biological samples [8] has the potential to become a valuable tool in the diagnosis and risk prediction of various diseases, including cancer, and can even reveal subtle abnormalities [9]. The importance of studying the metabolome to identify cancer biomarkers has already been demonstrated, and is an expanding field [10]. Specifically, studies of metabolites to identify cancer fingerprints were helpful to elucidate new therapeutic targets, such as CD147/EMMPRIN and CD44 in prostate cancer [11,12,13] and to predict prognosis [14]. The latter would be a major step forward for many patients with thymomas exhibiting “aggressive histotypes” but low tumor stages (e.g., B2 or B3 thymomas in stage pT1a/Masaoka-Koga stages IIa or IIb) [15], in whom over- or under treatment is a clinical issue, since it is currently unknown which patients would profit from adjuvant radiotherapy [16].

In terms of current treatment strategies, platinum-based chemotherapy is the standard first line systemic treatment if mostly curative resection with or without adjuvant radiotherapy is not possible in patients with advanced or metastatic TETs [16,17,18,19]. In contrast, no standardized salvage treatments are currently implemented if patients are resistant to platinum-based therapies [19,20]. However, recent clinical trials provided some promising data on the activity of new ‘target therapies’, particularly for patients with TCs [19]. These drugs include multikinase inhibitors with anti-angiogenic potential (e.g., sunitinib [21] and lenvatinib [22]), mTOR inhibitors (e.g., everolimus [23]) and immune checkpoint inhibitors (ICIs) (e.g., blockers of PD-1 [24,25] and PD-L1 [26]). Since ICIs unpredictively induce severe autoimmune adverse reactions in a high proportion of patients with TETs, particularly thymomas, use of ICIs should be considered only in the context of clinical trials [27].

Recent technological progress in NMR spectroscopy and mass spectrometry (MS), the two most accepted methods used to measure metabolites, has improved the sensitivity and spectral resolution of analytic assays. Although HRMAS ^1^H-NMR spectroscopy has a lower sensitivity than MS, it has the advantage of allowing easier quantification of metabolite signals, as no compound specific standards are required, and the technique is non-destructive. The latter aspect is relevant under the perspective of an ideal clinical workflow: as we outline below, conventional unfixed diagnostic needle biopies appear as optimal tumor material for immediate HRMAS ^1^H-NMR-based metabolite profiling followed by histopathology to determine conventional tumor characteristics in the very same biopsy. These advantages of NMR outweigh the higher sensitivity of MS [28,29]. Metabolite extracts, i.e., liquid samples from 2D cell culture, are not the only material accessible with NMR. With “High Resolution Magic Angle Spinning” (HRMAS) ^1^H-NMR [30], the identification and quantification of metabolites within intact tissue is feasible. Nevertheless, direct measurement of tissue samples by NMR spectroscopy is hampered by the broadening of the resonances due to effects, such as dipolar coupling, chemical shift anisotropy, and differences of bulk magnetic susceptibility. If, however, the sample is spun at the “magic angle” θ = 54.7°, where θ is the angle between the sample tube and the external magnetic field, two of these line broadening factors can be substantially reduced [30]. For the line narrowing to be successful, the spinning rate has to be larger than the strength of the underlying line broadening mechanism and is typically chosen as 5 kHz at 600 MHz [30].

Using this well-established [31,32,33,34,35] and validated [36] technology, we here compare metabolite profiles of a spectrum of indolent and aggressive TETs and identify enriched metabolic pathways through metabolite set enrichment analysis (MSEA). Since we investigate a rare form of cancer with only a small cohort of tumors available for analysis and no independent cohort of snap frozen tissue samples available for validation–implying that MSEA may be vulnerable to false positive results—we combine metabolic profiling for functional pathway elucidation with analysis of transcriptomic profiles of these pathways in a large, independent cohort of TETs (the TCGA database). These data were used to check whether the enriched pathways identified through metabolite profiling are accompanied by a comparable enrichment of identical but transcriptome-based metabolic (KEGG) pathways. As alterations in the transcriptome of cancer cells often reflect changes in the genome, transcriptional analysis can deliver information on the gene expression modifications of the tumor and may help elucidate new therapeutic targets for rare tumors. This strategy enabled us to include our new findings into already existing knowledge.

Therefore, the aim of the present study was to identify metabolic profiles across the major histological types of TETs and provide clues to new potential vulnerabilities of TETs. From our results, we conclude that validation of our findings in prospective studies with optimally obtained tumor material is warranted.

## 2. Materials and Methods

### 2.1. Patients and Tissue Samples

Patient characteristics are summarized in Table 1. Samples were classified according to WHO criteria [1]. Where appropriate, thymomas were grouped as indolent thymomas (WHO type A, AB and B1 thymomas) or aggressive TETs (B2 and B3 thymomas and TCs). The study was approved by the local ethics committee (approval 2018-516N-MA) at the Medical Research Center, Medical Faculty Mannheim, Heidelberg University, Mannheim, Germany, and by the ethics committee of the Leibniz-Institut für Analytische Wissenschaften—ISAS-e.V., Dortmund, Germany.

### 2.2. Sample Preparation and Measurement Conditions for NMR

Until preparation, the samples were stored at −80 °C for NMR analysis. Preparation of the samples was performed in a completely humidity free environment in a nitrogen atmosphere at −10 °C [30]. These conditions were essential in order to prevent any possibility of water condensation due to humidity changes, which occurs when working in an open environment on dry ice. After removing the tissue from −80 °C, it was kept at −10 °C for 30 min to allow its temperature to adapt to that of the surrounding. Tumor tissue samples were obtained using a 2-mm punch (PFM Medical, Cologne, Germany) so that samples could fit into 33-μL disposable inserts (DIs) (Bruker, Rheinstetten, Germany) for the 4-mm magic-angle spinning rotors. The tissue was weighed on a calibrated balance. Next, the DIs were filled with a solution containing the internal standard 3-trimethylsilylpropionic-2,2,3,3-d_4_-acid sodium salt (TSP) in D_2_O in order to calibrate the spectrum to 0.0 ppm.

NMR spectral acquisition was performed using a Bruker Avance III NMR spectrometer equipped with a 14.1 T magnet at 278 K. Acquisition and pre-processing of NMR spectra was performed under the control of a workstation with TopSpin 3.2 (Bruker BioSpin, Karlsruhe, Germany). Two different ^1^H-NMR spectra were collected: a 1D ^1^H spectrum providing quantitative metabolite data for statistical analysis, while 2D TOCSY and 2D ^1^H-^13^C HSQC experiments assisted in peak assignment and metabolite identification using standard Bruker pulse programs. For 1D ^1^H spectra performed for each sample, a Carr Purcell Meiboom Gill (CPMG) pulse sequence with 200 echoes was used to suppress the macromolecular background. Solvent signal suppression was achieved by presaturation during the relaxation delay (Frequency range 7 kHz, 16 K data points, relaxation delay 4 s, 128 scans, HRMAS rotation frequency 5 kHz). All spectra were processed using an exponentially decaying window function with a line broadening of 0.3 Hz and the baseline was automatically corrected. As already known, the TSP signal may be affected by proteins or other macromolecules present in the samples, and as a result we used the “Electronic REference To access In vivo Concentrations” (ERETIC) technique for the calibration of sample spectra [37]. The spectral region between 0.8 to 10 ppm was analyzed for metabolites and their relative concentrations (in mM) using the Chenomx NMR Suite 7.3 (Chenomx Inc., Edmonton, AB, Canada) based on its 600 MHz library. Chenomx NMR Suite 7.3 compares the integral of a known reference signal (e.g., TSP or formic acid) with signals derived from a library of compounds containing chemical shifts and peak multiplicities. In addition, the identification of selected metabolites was also cross checked from the Human Metabolome Database (HMDB) and from the literature.

A sample of a 0.3%-TSP-solution in D_2_O was used as the ERETIC reference, which was measured under the same conditions as the tissue samples. The reference signal was calibrated with ERETIC, imported into the Chenomx-Software and all metabolite concentrations were calculated in relation to this reference signal. From the resulting relative concentrations, the absolute concentrations in the tissue sample were calculated as follows:

$$\mathrm{Metabolite}\;\mathrm{concentration}=\frac{\mathrm{mass}\;\left(0.3{\%\;\mathrm{TSP}\;\mathrm{in}\;D}_{2}O\;\mathrm{in}\;\mathrm{mg}\right)\times 0.003\;}{\mathrm{molecular}\;\mathrm{mass}\;\mathrm{TSP}\;\left(172.24g/\mathrm{mole}\right)}$$

$$\times \frac{\mathrm{concentration}\;\mathrm{of}\;\mathrm{metabolite}\;\mathrm{signal}\;\mathrm{in}\;\mathrm{Chenomx}\;\left(\mathrm{mM}\right)}{\mathrm{concentration}\;\mathrm{of}\;\mathrm{TSP}\;\mathrm{from}\;\mathrm{ERETIC}\;\left(\mathrm{mM}\right)}\times \frac{10^{6}}{\mathrm{mass}\;\mathrm{of}\;\mathrm{tissue}\;\left(\mathrm{mg}\right)}$$

Statistical analyses were performed using the web server Metaboanalyst 5.0 (https://www.metaboanalyst.ca/MetaboAnalyst/ModuleView.xhtml accessed on 9 March 2022) [38], as well as R-scripts using the same procedures for a customized graphical representation, where sample specific normalization allowed the manual adjustment of relative concentrations based on biological inputs (i.e., volume, mass), and row-wise normalization allowed the general-purpose adjustment for differences among samples. Prior to normalization, described in detail in Section 2.3, imputing of missing values (set to zero in our raw data) was performed using one fifth of the smallest value measured for the respective metabolite in the sample set. Data transformation and scaling were accomplished using two different approaches to make features more comparable: Raw data were scaled using mean-centering and intensities in each spectrum were normalized to the sum of all metabolite concentrations of a given sample to avoid the contribution of dilution effects.

### 2.3. NMR Related Statistical Analysis

Multivariate statistical analysis, namely sPLS-DA [39], was applied to the metabolomic profile dataset to provide insights into the separations between the two groups. Since the mainly water-soluble metabolites are diluted to different extents in different tissues (typically due to different fat/lipid contents), normalization of metabolite concentrations (amounts per weight of tissue) is necessary. This was achieved by dividing individual metabolite concentrations by the sum of all metabolite values in the respective sample. Mean centering was applied to remove the offset from the data. In this way, one gets a reduced rank representation of the model and avoids numerical problems as a simpler model can be fitted to the data. Additionally, data scaling was done after mean centering of the data by dividing each value by the standard deviation of this metabolite concentrations over all measured samples. This procedure ensures that each metabolite is treated equally in the principal component analysis (PCA) disregarding absolute concentrations. Moreover, using normalized data without mean centering and scaling, AUC values obtained from receiver operating characteristic (ROC) values [40] were calculated to verify which metabolites had the highest sensitivity/specificity ratio for diagnosis. The aim of classical ROC curve analysis is to evaluate the performance of a single feature, e.g., a metabolite, as a biomarker (see below). To check whether there is a significant statistical difference between the indolent and aggressive groups of TETs, a Welch two sample *t*-test was run on the same data using the “*t*.test” module implemented in R 3.6.2 (http://www.r-project.org/ accessed on 9 March 2022).

### 2.4. MetPA Analysis of Metabolite Profiles for Metabolic Pathway Detection

To investigate whether identified metabolites represent a random sample of compounds or reflect the enrichment of known metabolic pathways, the identified metabolites and their concentrations were subjected to MetPA metabolic pathway analysis [41] using Metaboanalyst 5.0 (Xia Lab @ McGill university, Montreal, QC, Canada) [38]. The output of the MetPA analysis are KEGG pathways (henceforth called “KEGG metabolite-based metabolic pathways” [41]), *p*-values that indicate the degree of enrichment of these pathways, and ‘impact-values’. “Impact” is a quantitative measure of the importance of an individual metabolite in a given pathway: the higher the impact, the higher the functional relevance of the respective metabolite [41]. *p*-values from MetPA are multiple testing corrected [41].

### 2.5. Quantitative Real Time PCR

Total RNA was isolated from whole tissues using TRIzol reagent (Invitrogen Waltham, MA, USA) and treated with DNase using DNA-free™ DNA Removal Kit (Thermofisher scientific, Bremen, Germany) to remove any DNA contamination. mRNA was reverse transcribed with RevertAid™ H Minus Reverse Transcriptase (Fermentas, Hamburg, Germany) using the manufacturer’s protocol and the resulting cDNA was used for quantitative PCR. This procedure is henceforth called qPCR, which was performed in duplicate on a StepOnePlus^TM^ TaqMan PCR System (ABI, Applied Biosystems, Borken, Germany) using FAST SYBR Green master mix (ABI, Germany). Primer sequences and respective gene names are available in Tables S1 and S2, respectively. Post-PCR analyses were carried out automatically to check the dissociation or melting curves at the end of each PCR experiment to exclude primer–dimers and to determine the specificity of the PCR reaction and resulting product (Figure S1). The relative expression (RQ) of a given gene was calculated using the ΔΔCt method (also called 2^-deltadelta Ct method) in relation to the expression of the housekeeping gene, GAPDH. In some figures (e.g., Figures S2 and S3) relative expression in thymic tumors in our “Own tumor cohort” is given as “fold change” normalized to the relative expression in normal thymus. For this normalization, the mean relative expression in the normal thymus NT.1 in Table 1 was used.

### 2.6. Gene Set Enrichment Analysis (GSEA)

For GSEA, the RNAseq TCGA raw data was obtained from cBioportal (https://www.cbioportal.org) last accessed on 9 March 2022. The count data was transformed to log2-counts per million (logCPM) using the voom-function from the limma package [42]. Differential expression analysis was performed using the limma package in R. The ranking gene list was generated by sorting the results using the *t*-values obtained from differential expression analysis (limma package). GSEA pathway analysis was done with R and bioconductor using fgsea package [43] and the EnrichmentBrowser package [44] in R using the pathway information from KEGG data base (https://www.genome.jp/kegg/pathway.html) last accessed on 9 March 2022.

### 2.7. Transcriptomics Related Statistical Analysis

The statistical analyses of gene expression data were performed with GraphPad Prism V6.0 (GraphPad Software Inc, La Jolla, CA, USA). Two-tailed student’s *t*-test and one-Way ANOVA were applied when gene expression of the metabolism-related genes was altered in different groups of thymic tumors in comparison to normal thymi. A subsequent Tukey’s multiple comparisons test was used to compare variances, with *p* < 0.05 at a confidence level of 95% (*p* < 0.05) being considered as significant. 

## 3. Results

### 3.1. ^1^H NMR Spectroscopy Reveals 37 Metabolites in TETs

**Figure 1:**
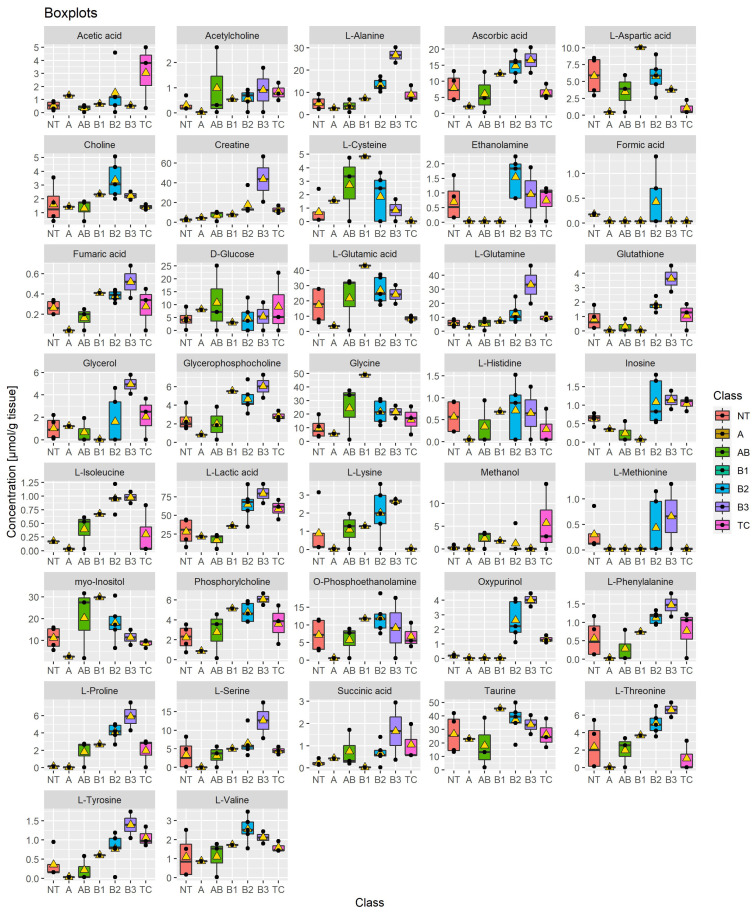
Boxplots showing the concentrations (*y*-axis) of the 37 metabolites found in normal thymi (NT, *n* = 4), thymomas (A, AB, B1, B2, B3, *n* = 12) and thymic carcinomas (TC, *n* = 3). The black bars show the respective median of a distribution, while the yellow triangles show the respective average. Each box is drawn from the 25th to the 75th percentile. Please note that the scale of the *y*-axis was adapted to the concentration range and is therefore different among the different metabolites.

**Figure 2:**
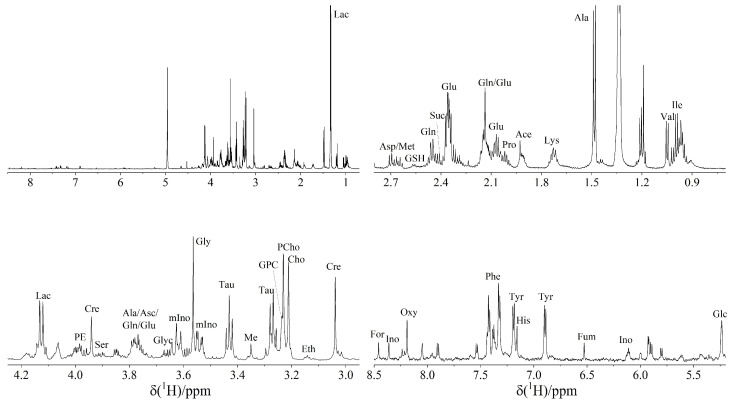
Representative 1D HRMAS ^1^H-NMR spectrum of a B2 thymoma measured at a tissue temperature of 4 °C and referenced to the internal standard, TSP (3-trimethylsilylpropionic-2,2,3,3-d_4_-acid sodium salt), full spectrum (upper left) and three expansions. Abbreviations: Ace: acetate, Ala: alanine, Asc: ascorbate, Asp: aspartate, Cho: choline, Cre: creatine, Eth: ethanolamine, For: formiate, Fum: fumaric acid, Glc: glucose, Glu: glutamate, Gln: glutamine, GSH: glutathione, GPC: glycerophosphocholine; Glyc: glycine, His: histidine, Ile: isoleucine, Ino: inosin, Lac: lactate, Leu: leucine, Lys: lysine, Met: methionine, mIno: myo-inositol; Oxy: oxypurinol, PCho: O-phosphocholine, PE: O-phosphoethanolamine, Phe: phenylalanine; Pro: proline, Ser: serine, Succ: succinate, Tau: taurine, Tyr: tyrosine, Val: valine.

Analysis of 15 snap-frozen TETs using HRMAS ^1^H-NMR spectroscopy revealed a total of 37 metabolites (Table S3), the concentrations of which are given in Figure 1. No single sample contained all 37 metabolites: 3 cases (all aggressive TETs) showed 33 metabolites, 2 cases (1 indolent, one aggressive TET) showed 32 metabolites, 2 cases (1 indolent, 1 aggressive TET) showed 31 metabolites, while 8 cases (3 indolent, 5 aggressive TETs) showed less than 31 metabolites. Overall, more metabolites could be detected in the aggressive TETs compared to the indolent TETs. A representative HRMAS ^1^H-NMR spectrum is shown in Figure 2.

### 3.2. Metabolic Profiles Are Closely Associated with WHO Histotypes

**Figure 3:**
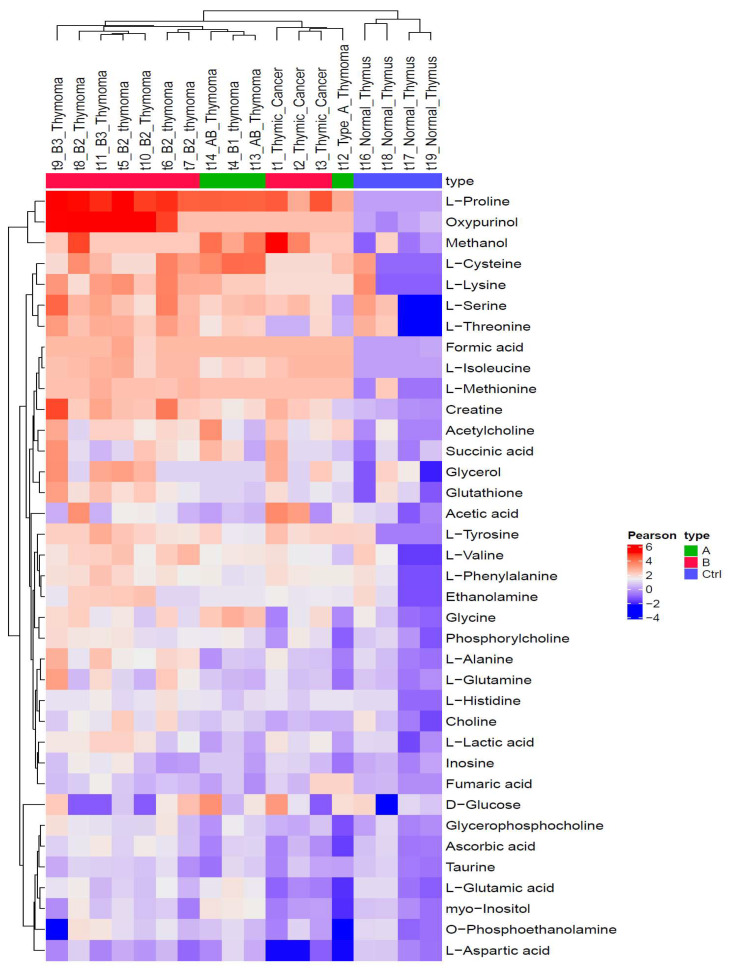
Heatmap with identified metabolites in thymic epithelial tumors (TETs): Hierarchical clustering analysis (HCA) was performed on 37 standardized, log2-transformed values of metabolites from 14 of the 15 TETs (1 A, 3 AB, 1 B1, 5 B2, 2 B3 thymomas and 3 thymic carcinomas, TC) and 4 non-neoplastic thymi (NT). One AB thymoma was exempt from the analysis since all metabolite concentration were extremely low or even zero, resulting in graphical over representation of this sample. To create the heatmap, the values of the raw data from Table S4 were log2 transformed and then the value of each metabolite was standardized (subtracted) with the respective mean value of the log2 transformed values of the normal thymi. Note that aggressive thymomas (B2, B3 thymomas) and TCs form distinct clusters. Note: t1, t19, t3, etc. are the internal labels of the samples as used in the excel sheet with the metabolite levels as provided in the Supplement Materials (Table S4).

Unsupervised cluster analysis of metabolite expression profiles of 14 of the 15 cases (from Table S4) are shown in the heatmap in Figure 3. TCs and aggressive (B2 and B3) thymomas each form one group. A third group consists of the indolent thymomas (A, AB, B1). All TETs cluster separately from non-neoplastic thymi.

One striking finding was the higher concentrations of specific metabolites in the aggressive, i.e., B2 and B3 thymomas compared to TCs, including proline, alanine, oxypurinol, choline and cysteine (Figure 1). Low levels of cysteine were characteristic of TCs, although single B2 and B3 thymomas showed low levels as well. Taking the similarity between B3 thymomas and TCs in terms of their shared dominance of tumor cells over rare T cells into account (Table 1), the much lower levels of several metabolites in TCs (e.g., alanine, glutamine, glutathione, proline, serine and threonine) are particularly remarkable, since they are likely related to the actual tumor cells and may have tumor biological relevance. For example, low levels of alanine have been observed in various cancer types [45], to which we can now add TCs. While the mechanisms leading to low alanine levels are poorly understood [45], they were associated with apoptosis resistance in melanoma [46] and high aggressiveness in various non-thymic carcinomas [45], which fits well with the particularly high apoptosis resistance and clinical aggressiveness of TCs [1,3]. Interestingly, there was no metabolite with a consistently higher level in TCs than in B2 and B3 thymomas; even lactic acid levels that were higher in aggressive (B2, B3 and TC) than indolent TETs were lower in TCs than in B2 and B3 thymomas (Figure 1). Furthermore, succinic acid and L-isoleucine were found in 13 and 11 samples, respectively, but were not detectable in any non-neoplastic thymus.

### 3.3. HRMAS ^1^H-NMR Analysis Discriminates ‘Indolent’ from ’Aggressive’ Groups of TETs

When we compared the clinically important groups of indolent (*n* = 5) and aggressive (*n* = 10) TETs, only 7 of the 37 metabolites showed significantly different levels (*p* < 0.05; Welch two sample *t*-test) (Table S3 and Figure S4). Higher levels of cysteine and myo-inositole were typical of indolent TETs, while higher levels of alanine, glutathione, inosine, lactic acid and oxypurinol were characteristic of the group of aggressive TETs.

**Figure 4:**
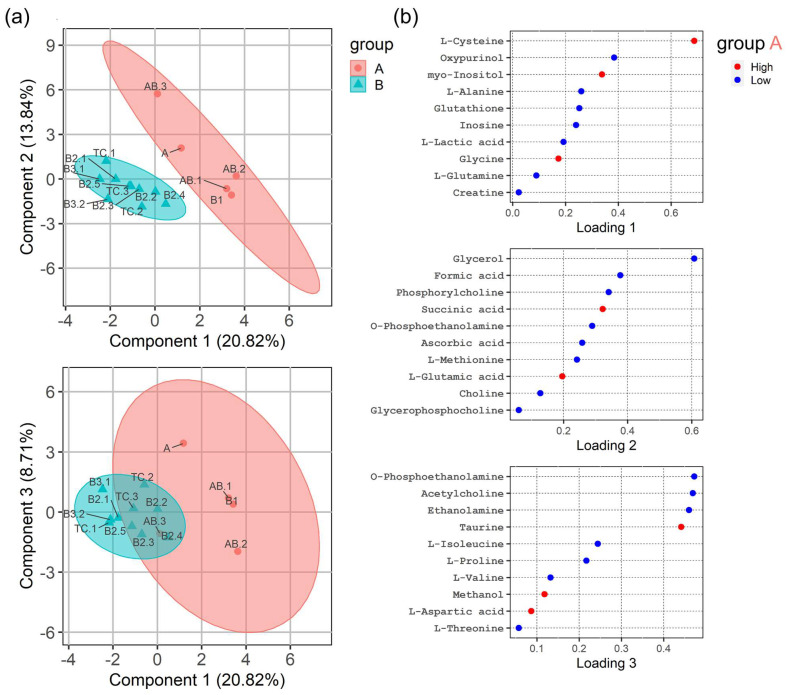
(**a**) sPLS-DA scores plot (3 components) showing clustering between indolent (group A) and aggressive (group B) thymic epithelial tumors (TETs). 95% confidence intervals are given in red (indolent group) and turquois (aggressive group). Top: Component 1 against component 2. Bottom: Component 1 against component 3. (**b**) sPLS-DA loadings plots for components 1 (top), 2 (middle) and 3 (bottom) showing upregulation and downregulations of metabolites for indolent TETs (upregulation is shown in red, downregulation in blue). The naming of the triangles and dots refers to the different tumor cases given in Table 1, for example B2.3 means the third B2 thymoma case from the top as listed in Table 1.

To investigate whether a broader spectrum rather than a small set of metabolites was more suitable to separate indolent from aggressive TETs, a “scarce partial least square-discriminant analysis” (sPLS-DA) was calculated. As shown in Figure 4, high concentrations of cysteine, myo-insositol, glycine, glutamic acid and glucose contributed to the delineation of the group of indolent TETs, while the group of aggressive TETs was characterized by higher concentrations of alanine, glutathione, inosine, lactic acid, ascorbic acid, creatine, inosine, ethanolamine, fumaric acid, glutamine, isoleucine, phosphoethanolamine, proline, serine, tyrosine, phosphocholine, phenylalanine and glycerophosphocholine. Seven metabolites, namely alanine, cysteine, glutathione, inosine, lactic acid, myo-inositol and oxypurinol showed significantly different levels between indolent and aggressive groups of TETs (*p* < 0.05) (Table S3), and nine metabolites (creatine and glutamine in addition to the aforementioned 7 metabolites) showed an area under the curve of >0.8 when receiver operating characteristic (ROC) curves [40] were generated (Table S5).

### 3.4. HRMAS ^1^H-NMR Analysis Reveals Differentially Activated Metabolic Pathways in TETs

The complete set of metabolites (Table S4) of the 15 TETs was subjected to a MetPA metabolic pathway analysis [41] to determine whether the identified metabolites reflect the activation of distinct KEGG metabolic pathways. As shown in Table 2, MetPA analysis revealed six “KEGG metabolite-based metabolic pathways” that were significantly activated in the group of aggressive TETs. In functional terms, these pathways may have an impact on trans-sulfuration, homocysteine and tricarboxylic acid (TCA) cycles, the management of reactive oxygen species (ROS) and glycolysis (Table 2), i.e., on cellular processes relevant to tumor biology. Surprisingly, MetPA analysis failed to reveal a single activated metabolic pathway in the group of indolent TETs.

### 3.5. Metabolic Gene Set Enrichment Analysis of the TCGA Transcriptomic Dataset

**Figure 5:**
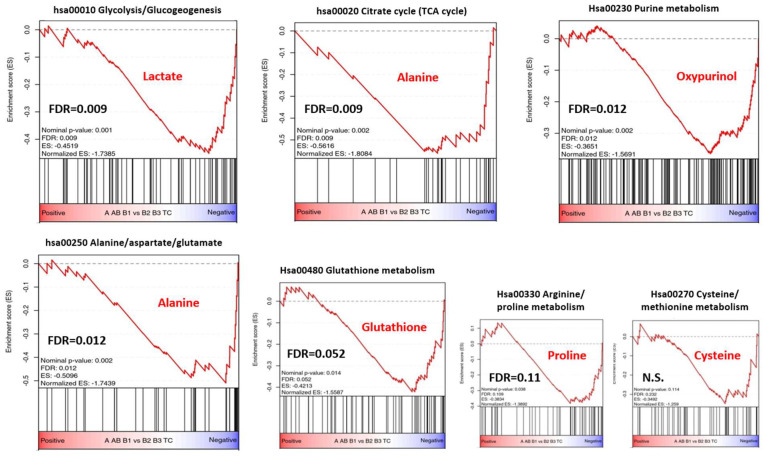
Gene set enrichment analysis (GSEA) of the indicated KEGG pathways comparing transcriptomic profiles of the group of indolent (A1, AB, and B1 thymomas) and aggressive thymic epithelial tumors (TETs) (B2 and B3 thymomas and thymic carcinomas, TC) as extracted from the TCGA, PanCancer Atlas dataset. Corresponding “key metabolites” identified in the HRMAS ^1^H-NMR-based analysis (Table 2) are given in red. Alanine as “key metabolite” was assumed to be functionally linked to two KEGG pathways: The TCA cycle and the alanine/aspartate/glutamate pathway. In addition to the Hsa00270 Cysteine/methionine pathway, the Hsa00260 Glycine/serine/threonine pathway (not shown) was also not enriched. Of note, the selection of the purine metabolism pathway in relation to high oxyurinol levels is only a hypothesis, since it is unknown whether oyxpurinol is an endogenous metabolite of purine metabolism or derived from an unknown food ingredient (we excluded allopurinol as a source of oxypurinol in the respective patients through clinical history). FDR, false detection rate.

To investigate whether the KEGG metabolite-based metabolic pathways (Table 2) were reflected by corresponding enriched KEGG pathways at the transcriptomic level (henceforth called “KEGG transcriptome-based metabolic pathways”), we used gene set enrichment analysis (GSEA) [43] and the DAVID pathway overrepresentation tool [47] to interrogate the transcriptomic profiles of the TCGA thymic cancer database (CbioPortal, https://www.cbioportal.org accessed on 9 March 2022) that contains 10 type A, 48 AB, 12 B1, 25 B2, and 10 B3 thymomas, as well as 10 TCs [4]. The comparison between the two groups of indolent (A, AB, B1 thymomas) and aggressive TETs (B2 and B3 thymomas and TCs) by ranking genes according to their t-values for gene set enrichment analysis (GSEA [43]) revealed—in accordance with findings in Table 2—that pathways related to lactate (glycolysis), alanine (the TCA cycle and the alanine/aspartate/glutamate pathway) and gluthathione showed significant or close to significant enrichment, while the cysteine/methionine pathway (Figure 5) and the glycine/serine/threonine pathway (not shown) were not significantly enriched. Due to the paucity of associated genes (*n* = 5), GSEA was not applicable for the D-Glutamine/D-Glutamate pathway. Since MetPA analysis (Table 2) did not identify pathways linked to the “high-level metabolites“, oxypurinol and proline (Figure 3), we selected the KEGG purine metabolism pathway (hsa00230) for oxypurinol (although hsa00230 does not explicitly list oxypurinol as metabolite), and the arginine and proline pathway (hsa00330) for proline as hypothetical candidates for GSEA. While the purine pathway was significantly enriched (FDR = 0.012), the arginine/proline pathway showed only a trend (FDR = 0.11) (Figure 5).

To check the GSEA results with an alternative method, we next investigated the TCGA TET cohort with the DAVID annotation database provided at https://david.ncifcrf.gov (last accessed on 9 March 2022.) to identify pathway overrepresentation [47]. This analysis was focused on 90 genes mentioned in (Table S2) selected from the “KEGG transcriptome-based metabolic pathways” from Figure 5 on the basis of their most differential expression between indolent and aggressive TETs. Of note, the 90 genes included 53 genes that showed significantly different expression (with *p* < 0.05) between individual thymoma types with similar lympho-epithelial composition but different (indolent versus aggressive) biological behavior (as exemplified by the comparison between type A and B3 thymomas) (Table S7). This established strategy considers the fact that the variable abundance of non-neoplastic lymphocytes in the various TET histotypes (Table 1) can obscure molecular differences between them [4]. The overrepresented pathways identified in this way are given in Table 3 and overlap with the significantly enriched pathways found through GSEA (Figure 5). In addition, the arginine/proline pathway and–at lower significance levels–the cysteine/methionine and the glycine/serine/theronine pathways were also found to be overrepresented in the group of aggressive TETs (Table 3). 

Finally, we investigated whether the TCGA cohort (*n* = 115) might be representative of our TET cohort analyzed by NMR (*n* = 15). To this end, we used qPCR to quantify transcripts of 28 genes selected from the identified metabolic pathways (Table 3) in whole RNA extracts of 51 TETs from Mannheim (including the 15 cases analyzed by NMR) as described previously [3]. Since the relative expression profiles of up to 26 of the 28 metabolism-associated genes were similar in both cohorts (Figures S2 and S3, Table S6), we assume that the findings obtained with the TCGA dataset are of relevance to our cohort of TETs.

## 4. Discussion

This first ever metabolomics study of TETs using HRMAS ^1^H-NMR has provided four important findings: (1) 37 metabolites were detected in the major TET subtypes, (2) metabolite-based cluster analysis distinguished the three clinically relevant TET subgroups-indolent and aggressive thymomas and TCs, (3) metabolite-based pathway analysis gave hints to activated metabolic KEGG pathways shared between aggressive thymomas and TCs, and (4) differentially activated metabolic pathways identified through metabolite profiling were generally also enriched at the transcriptomic level in the groups of indolent versus aggressive TETs in the TCGA TET dataset [4].

### 4.1. General versus TET-Specific Metabolites

**Figure 6:**
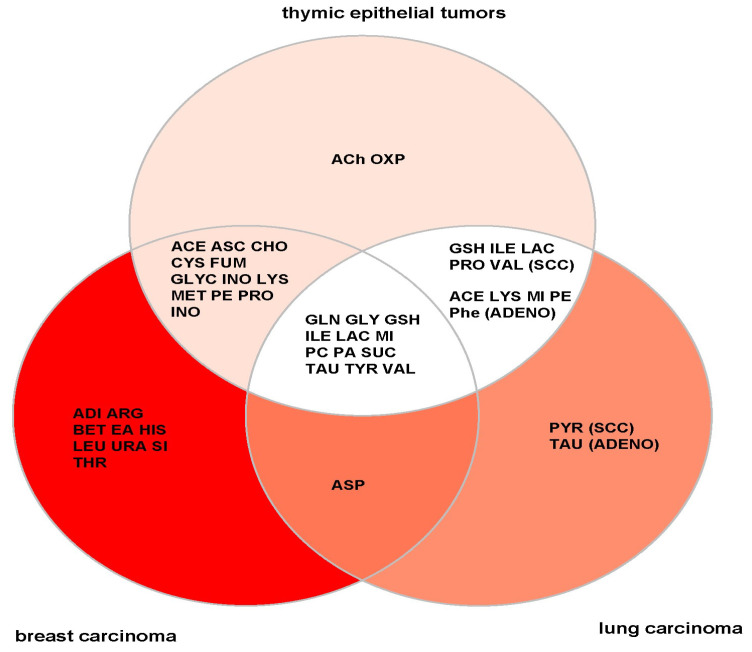
Venn diagram illustrating the metabolite distribution in breast cancer [48], thymic epithelial tumors (TETs) (this study) and lung cancer [55]. Note that most detected metabolites in TETs were also present in lung and breast cancers. Only acetylcholine (ACh) and oxypurinol (OXP) are ‘unique’ metabolites of TETs. The ‘unique’ metabolites of squamous cell (SCC) lung carcinoma and adenocarcinoma (adeno) of the lung were pyruvate (PYR) and taurine (TAU), respectively.

The 37 metabolites detected here in a spectrum of TETs compares well with the number of metabolites (9–34) reported in previous studies of non-thymic cancers [48,49], including 42 metabolites detected recently using the same HRMAS ^1^H-NMR technique in a cohort of breast cancers [50]. Although breast cancers in contrast to TETs are mostly hormone responsive adenocarcinomas with very different genetic aberrations, the overlap of detected metabolites between the two cancer types was extensive (Figure 6). This suggests that most detected metabolites are involved in cancer-related processes, and are not specific to a particular organ or tumor type. The former is exemplified by the higher levels of lactic acid in aggressive thymomas and TCs, which likely reflect the switch in energy metabolism known as the Warburg effect that is observed in many cancers [51]. Similarly, high levels of glutathione, one of the key regulators of the cellular redox state, are also typically found in a variety of aggressive cancers and associated with tumor progression and increased resistance to chemotherapy [52]. On the other hand, the low levels of cysteine and myo-inositol observed in the group of aggressive TETs are also typical of aggressive non-thymic solid and hematopoietic cancers and have been linked to alterations in the redox state and oncogenic PI3K/AKT signaling, respectively [53,54]. In addition, low levels of cysteine and glutamic acid in the aggressive TETs fit very well with the observed elevated levels of glutathione, because both metabolites are glutathione precursors.

In contrast to several non-specific metabolic features detected in TETs, acetylcholine and oxypurinol are potentially TET-specific metabolites since they were undetectable in breast and lung cancers (Figure 6). The detection of appreciable levels of acetylcholine is intriguing since acetylcholine receptor subunits are expressed in the tumor cells of thymomas, particularly if associated with Myasthenia gravis (MG) [56]. Accordingly, sufficiently powered, future metabolite profiling analyses of myasthenic versus non-myasthenic TETs might open new insights into the enigmatic pathogenesis of TET-associated MG. Why acetylcholine was detected in TETs but not in breast and lung cancers is unclear. Acetylcholine levels in tissues are regulated by the interplay of synthesis, degradation and transport [57]. Therefore, we analyzed TCGA transcriptomic data sets of TETs, breast and lung cancers (CBioPortal, http://www.cbioportal.org/ accessed on 9 March 2022) for the expression of the two key synthesizing enzymes (choline acetyltransferase [CHAT] and carnitine acetyltransferase [CRAT]), the key degrading enzyme (acetylcholine esterase [ACHE]) and several transporters (including the organic cation transporters, OCT1-3 [SLC22A1-3]) but did not find a unique RNA expression pattern that could easily explain the higher levels in TETs (Figure S5) [58]. To validate and eventually explain the observed difference, future studies could simultaneously quantify acetylcholine in TETs, breast and lung cancer biopsies using HRMAS ^1^H-NMR, as well as a complementary technique (e.g., HPLC) to exclude biases due to the technique. This, however, is unlikely since breast tumors were analyzed using the identical HRMAS ^1^H-NMR technique [30] and no acetylcholine was detected. In addition, acetylcholine metabolizing enzymes and transporters could be analyzed at the protein level, since their expression and thus their function may not exclusively be regulated through transcription [58,59].

The occurrence of oxypurinol in aggressive TETs (Figure 1) is even more enigmatic. While careful anamnesis excluded allopurinol medication as a source of oxypurinol in our patients [60], high natural levels of intratumorous oxypurinol have not been reported thus far, and there is no known physiological connection between oyxpurinol and the purine metabolism. Nevertheless, we observed enrichment of the KEGG purine pathway in the group of aggressive TETs (Figure 5) with overexpression of three purine metabolism-associated genes, *ADSL*, *HPRT1* and *IMPDH1* (Table 3) [61,62]. Interestingly, ADSL one of the enzymes involved in purinosome complex formation and de novo purine synthesis [63] showed high expression in our cohort of B2 and B3 thymomas but not in TCs (Table 3; Figure S2). Since ADSL is a known oncogenic driver in several cancers and a potential predictive biomarker for response to the purine antimetabolite, 6-mercaptopurine in preclinical models [64], a more in-depth analysis of ADSL, especially in B2 and B3 thymomas appears warranted under a therapeutic perspective. In summary: although purine metabolism appears as a potentially interesting oncogenic pathway in TETs (that needs validation in independent cohorts of TETs), it remains enigmatic how the oxypurinol detected in aggressive TETs is generated and whether it is formed inside the tumors themselves. However, since purine metabolites are constituents of tumor-derived exosomes [65] and oxypurinol was recently detected in the blood of non-thymic cancer patients [66], future studies should address the levels of oyxpurinol in TETs and the blood of TET patients with fully monitored medication. Furthermore, future in vitro studies may eventually help to clarify the source of oxypurinol. For instance, if long-term production of oxypurinol could be observed in ex-vivo TET cultures, this would argue for a natural source, while rapidly declining oxypurinol levels in vitro would challenge this argument.

### 4.2. Metabolite Profiles Meet KEGG Pathway and Gene Expression

Although the relatively low sensitivity of HRMAS ^1^H-NMR [30] explains the detection of only 37 metabolites, and not all 37 found in each investigated TET, the metabolite spectrum identified was sufficient to distinguish the three different TET groups by unsupervised cluster analysis: (i) the prognostically favorable type A, AB and B1 thymomas that can mostly be cured by surgery; (ii) type B2 and B3 thymomas that often require (neo-)adjuvant treatment concepts, have an unfavorable prognosis and commonly show paraneoplastic MG; and (iii) the often lethal TCs that are mostly unrelated to autoimmunity [1]. 

Furthermore, on the basis of the 37 metabolites, the MetPA algorithm was able to identify differentially activated metabolic pathways in the clinically most relevant group of aggressive TETs, i.e., B2 and B3 thymomas and TCs. The possible relevance of these differentially activated pathways was underpinned through gene set enrichment analysis (GSEA) of the independent TCGA TET dataset [4]. By GSEA, the glycolysis/gluconeogenesis pathway (together with the citrate/TCA cycle) was the most strongly enriched pathway in aggressive TETs (Figure 5). This is in line with changes expected in conjunction with an active Warburg effect-high levels of lactic acid, and known preferential expression of the glucose transporter GLUT1 in TCs and B3 thymomas [67]. While these features underlie the high power of 18F-fluorodeoxy-glucose-based positron emission tomography to predict aggressive TETs [68], they are again not specific for TETs but also encountered in many non-thymic aggressive tumors [69]. The same low specificity may also apply to the observed enrichment of the TCA cycle pathway and the alanine/aspartate/glutamate metabolic pathway (Figure 5) that are both typical of aggressive cancers [70].

### 4.3. Therapeutic Perspectives

Due to the differential expression of metabolites in TETs versus normal thymi (Figure 1), pathways related to proline, cysteine, glutathione, lactate and glutamine appear as potential therapeutic targets in refractory TETs, in which targeted treatments have remained elusive [71].

In agreement with the high levels of proline in aggressive TETs (Figure 3), we observed differential transcription of the *PYCR1* gene encoding Pyrroline-5-carboxylate reductase 1 in the TCGA dataset (Table 3, Table S7 and Figure S2). PYCR1 catalyzes the last step of proline biogenesis [72]. Since proline has a major impact on energy metabolism, glycolysis, the redox state, apoptosis and proliferation of tumor cells, PYCR1 has been suggested as a therapeutic candidate target in many cancers [72,73,74] to which we now can add aggressive TETs. PYCR1-targeting agents that block proline synthesis are in the early stages of development [75]. 

Low levels of cysteine (a glutathione precursor), in conjunction with high levels of glutathione (Figure 1) observed in TETs are reminiscent of the altered redox landscape reported in other cancer types that promotes tumor progression and treatment resistance [76]. This redox state is characterized by increased levels of both reactive oxygen species (ROS) and anti-oxidants like glutathione, thereby maintaining redox homeostasis and promoting tumor cell survival [77]. Drugs tipping the redox balance towards increased oxidative stress or towards reduced glutathione levels and synthesis, either directly or through cysteine starvation [78] are currently being developed [79] and appear worth testing in future studies.

High lactate levels (Figure 1), the enrichment of the glycolysis pathway (Figure 5), including overexpression of hexokinases (*HK1, HK3*) and pyruvate kinase (*PKM*) (Table 3), as well as the high protein expression of the glucose transporter, GLUT1 [67] all hint to an active ‘Warburg effect’, and provide promising potential therapeutic targets for aggressive TETs [80]. Small molecule inhibitors to GLUT1, hexokinases and PKM2 are already available for preclinical testing [80]. Finally, a prominent ‘Warburg effect” implies that the cancer cells have a critical need to fuel the TCA cycle with glutamate through glutaminolysis for the synthesis of vital cellular constituents, including nucleotides and glutathione [79]. High levels of glutamine (Figure 1) and an activated glutamine/glutamate pathway (Table 3) suggest that this pathway is functional in aggressive TETs. Therefore, inhibitors of glutaminolysis and of the downstream TCA cycle appear as rational therapeutic strategies. In fact, Glutaminase (GLS) inhibitors, such as CB-839, an orally available, potent, and specific inhibitor of GLS, have shown anti-tumor efficacy. CB-839 disrupts the conversion of glutamine to glutamate and alters a number of downstream pathways, including the TCA cycle, glutathione production, and amino acid synthesis [81]. Therapeutically targeting the TCA cycle function could also be an attractive strategy to treat TETs. Targeting IDH1 (isocitrate dehydrogenase 1) that is overexpressed in aggressive TETs (Table 3 and Table S6) using IDH1 Inhibitors such as GSK864 as reported in preclinical tests in glioblastomas could be a new therapeutic option, especially when used in combination with inhibitors of RTK-PI3K signaling [82].

### 4.4. Limitations of the Study

Major limitations of our study are the comparatively small sample size, the heterogeneity of histotypes and the lack of an independent set of TETs to validate our HRMAS ^1^H-NMR results. In particular, we are aware of the fact that that our results must be interpreted cautiously, since MESA and GSEA analyses are prone to yield false positive results in the face of relatively low case numbers. These weaknesses are related to the extreme rarity of TETs, particularly TCs [1], and the even rarer chance to retrieve native tumor material for fast snap-freezing that is a prerequisite for HRMAS ^1^H-NMR. In future studies, core needle biopsies may be a promising approach to prospectively obtain tumor material of “near-to-in-vivo” quality. Although the combined use of innovative metabolite profiling in a small cohort of TETs and the analysis of transcriptional profiles of the same and related pathways in our own cohort of TETs, as well as the large, independent TCGA cohort of high quality TETs gave us new insights into their metabolic characteristics, it is necessary to validate our preliminary results in prospective, much larger collections of optimally retrieved tumor material (e.g., through core needle biopsies).

## 5. Conclusions

We conclude that HRMAS ^1^H-NMR is a valuable tool that provided new insights into the tumor biology of TETs, particularly the heterogeneity of their metabolic features that segregated well with clinical risk groups, i.e., indolent and aggressive thymomas and thymic carcinomas. While transcriptomic approaches helped validate most of the activated pathway that were identified by metabolite-based profiling of TETs, HRMAS ^1^H-NMR also identified “TET-specific” metabolites (such as acetylcholine), whose occurrence could not be readily explained by transcriptomic data. Therefore, we conclude that HRMAS ^1^H-NMR will deepen our understanding of TETs by revealing features that may be missed when using genomic techniques alone. The metabolite-derived findings reported here suggest that metabolic pathways that are commonly altered in many other cancer types are also affected in TETs, as exemplified by an active Warburg effect and glutaminolysis. Such abnormally activated pathways can compromise treatments (e.g., chemotherapy and immunotherapies) that are also currently used in inoperable TETS but have limited efficacy [83,84,85]. Therefore, the current findings–if confirmed in larger, independent cohort s of TETs–support the new perspective that targeting specific metabolic vulnerabilities of TETs may have the potential to improve the efficacy of current standard therapies and the prognosis of patients with aggressive TETs.

## Figures and Tables

**Table 1:** Clinico-pathological characteristics of the 12 thymoma patients and 3 thymic carcinoma (TC) patients, as well as 4 controls (non-neoplastic thymi, NT) studied here. Diagnosis: the histological classification of thymomas (types A, AB, B1, B2, and B3) and TCs followed the World Health Organization (WHO) [1]. Stage (I–IV): local tumor extension (I–III) and pleural spread (IVa) are given according to the Masaoka-Koga classification [15]. Lymphocyte contents of tumor samples were estimated in hematoxylin and eosin-stained representative histological sections as percentages of nucleated cells (in 10% increments) as described previously [4]. [MG status: presence (+) or absence (−) of Myasthenia gravis; *N*: number of cases; n.k.: not known].

Diagnosis	*N*	Age (Years)	Sex (M/F)	MG Status	Stage (I–IV)	Lymphocyte Content (%)
Type A	1	77	M	−	n.k.	10
Type AB	3 (AB.1–AB.3) *	46 77 77	M M M	− + −	II II II	30 60 30
Type B1	1	72	M	−	III	90
Type B2	5 (B2.1–B2.5) *	75 73 51 72 73	F F M F F	+ + − − n.k.	III III IVa IVa III	60 60 80 70 80
Type B3	2 (B3.1 and B3.2) *	80 58	F M	− −	I II	10 20
TC	3 (TC.1–TC.3) *	75 69 78	M M M	− − −	III II II	10 10 10
NT	4 (NT.1–NT.4) *	27 ** 42 39 20	M M *** F ** F *	− − + +		90 90 90 90

* Annotation of individual cases as used in the text; ** NT.1: qPCR-based relative gene expression values of NT.1 were used to normalize respective gene expressions in thymomas, TCs and other thymuses; *** NTs with thymitis (lymphofollicular hyperplasia).

**Table 2:** “KEGG metabolite-based metabolic pathways” identified by the MetPA algorithm using 37 metabolites detected in the groups of indolent (A, B1 and AB thymomas) and aggressive (B2, B3 thymomas and thymic carcinomas, TC) groups of thymic epithelial tumors (TETs). The first column lists the “key metabolite” associated with each enriched metabolic pathway (columns 2). The assumed biological function associated with the given pathway is listed in column 3. *p*-values represent the differences in enrichment of the MetPA-derived “metabolite-based metabolic KEGG pathway” (column 2) between indolent and aggressive groups of TETs. “Impact” is a quantitative measure of the importance of an individual metabolite in a given pathway: the higher the value, the higher the impact and the functional relevance of the individual metabolite in the respective pathway [41].

Key Metabolite	MetPA-Derived KEGG Pathway	Assumed Function	*p*-Value	Impact
Cysteine	Cysteine/methionine metabolism	Transulfuration Pathway	<0.001	0.22
Glycine	Glycine, serine and threonine metabolism	Homocysteine cycle	0.012	0.46
Glutathione	Glutathione metabolism	Redox state	0.013	0.37
Alanine	Alanine, aspartate and glutamate metabolism	TCA cycle	0.022	0.54
Lactate	Pyruvate metabolism	Glycolysis	0.029	0.14
Glutamine	Glutamine/glutamate metabolism	Glutaminolysis	0.032	0.50

**Table 3:** “KEGG transcriptome-based metabolic pathways” retrieved from the TCGA thymic epithelial tumor (TET) database [4] applying the DAVID overrepresentation analysis tool to 90 genes with significantly (*p* < 0.05) different expression between indolent TETs (A1, AB, and B1 thymomas) and aggressive TETs (B2, B3 thymomas; TCs).

Key Metabolite(s)	KEGG Pathways	KEGG-ID	*p*-Value	Fold Enrichment	FDR	Genes *
Glutathione	Glutathione metabolism	hsa00480	1.3 × 10^−^^9^	25.3	1.9 × 10^−8^	*GSTK1, GCLC, GGT6, GPX4, ANPEP, IDH1, 2, MGST1, MGST2, LAP3*
Alanine	Alanine, aspartate and glutamate metabolism	hsa00250	1 × 10^−7^	28.7	1.1 × 10^−11^	*ALDH4A1, ADSL, FOLH1, GOT1, RIMKLA, NIT2, ASS1*
Proline	Arginine andproline metabolism	hsa00330	2.9 × 10^−11^	28.7	5.8 × 10^−10^	*ALDH4A1, GOT1, P4HA1, NOS2, NOS3, ARG1, PYCR1, LAP3, PRODH, ALDH9A1*
Glucose and lactic acid	Glycolysis/Gluconeogenesis	hsa00010	5.3 × 10^−6^	15	3.9 × 10^−5^	*HK3, PKM, ALDOA, FBP1, PCK2, HK1, ALDH9A1, SLC2A1, SLC16A3, SLC16A14*
Oxypurinol	PurineMetabolism **	hsa00230	4.8 × 10^−^^4^	89.6	3.2 × 10^−3^	NT5C3A, ADCY10, ADSL ***, PKM, PNP, IMPDH1, NUDT2, HPRT1, DCK, GART
Cysteine	Cysteine and methionine metabolism	hsa00270	2.1 × 10^−^^3^	15.1	1.1 × 10^−3^	*SDS, GOT1, MDH1, CBS, SLC1A5, SLC3A2*
Glycine	Glycine, serineand threonine metabolism	hsa00260	2.3 × 10^−^^3^	14.7	1.1 × 10^−3^	*SDS, SHMT2, CBS, PSPH*

* Representative genes for each metabolic pathway are highlighted in bold. ** The assignment of Oxypurinol to “purine metabolism” is a hypothesis, since it is unknown, whether oyxpurinol is a metabolite that is involved in the purine pathway or is the derivative of an unknown food component other than allopurinol that was excluded here as source of oxypurinol through clinical history. *** increased transcription of *ADSL* (encoding adenylosuccinate lyase) in B2 and B3 thymomas.

## Data Availability

All data used and/or analysed during the current study are available from the corresponding authors.

## Supplementary Materials

The following supporting information can be downloaded at: https://www.mdpi.com/article/10.3390/cancers14061564/s1, Figure S1: Melting curve analysis of DNA duplexes demonstrating the specificity of some of the primers used in this study; Figure S2: Relative gene expression levels of 7 representative genes in our “Own tumor cohort” and the “TCGA tumor cohort; Figure S3: Relative gene expression levels of 21 additional representative genes in our “Own tumor cohort” and the “TCGA tumor cohort”; Figure S4: Concentrations of metabolites in the groups of clinically indolent and aggressive thymic epithelial tumors: Figure S5: Comparative gene expression profiles (RNA) of genes related to acetylcholine (ACH) metabolism across the TCGA datasets of thymic epithelial tumors (THYM), breast cancers (BRCA) as well as lung squamous (LUSC) and lung adenocarcinomas (LUAD) (CBioPortal); Table S1: Primers for quantifying mRNA levels of 28 selected genes in the thymic epithelial tumors that were studied here by HRMAS ^1^H-NMR; Table S2: Official full names of the 90 genes used for functional annotation clustering; Table S3: Metabolites detected in 15 snap-frozen thymic epithelial tumors using HRMAS ^1^H-NMR spectroscopy; Table S4: Metabolite concentrations in the 15 thymic epithelial tumors (TETs) and 4 non-neoplastic thymi (NTs) as determined by HRMA ^1^H-NMR spectroscopy (links to relevant Excel files); Table S5: Area under the curve (AUC) values obtained from receiver operating characteristic (ROC) curves analysis of the metabolite levels in the 15 thymic epithelial tumors (TETs) analyzed in this study; Table S6: *p*-values and significance levels calculated for the relative expression levels of 28 genes in our Own tumor cohort (Mannheim) and the TCGA tumor cohort; Table S7: Comparison of relative gene expression levels between indolent and aggressive thymic epithelial tumors taking into account their lymphoid cell content.
