# Supplementary material for: Metabolic Profiling of Thymic Epithelial Tumors Hints to a Strong Warburg Effect, Glutaminolysis and Precarious Redox Homeostasis as Potential Therapeutic Targets
Source: Cancers (Basel). 2022 Mar 18;14(6):1564. doi: 10.3390/cancers14061564 (PMC8945961; doi:10.3390/cancers14061564)
Supplement: Supplementary file 1 [file cancers-14-01564-s001.zip › Supplementary Figures Updated new S1-S5.pdf]

Supplementary Figures S1–S5

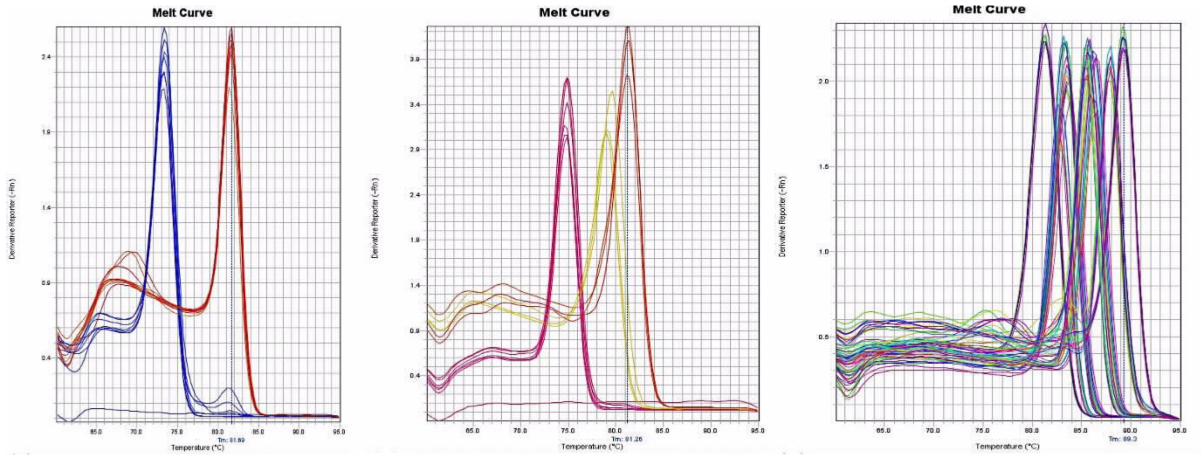

Figure S1. Melting curve analysis of DNA duplexes demonstrating the specificity of the primers used in this study (Table S1) for the quantification of the expression of the following genes of interest: Hexokinase 1 and 2 (in the left image), GAPDH, PKM, and PYCR1 (in the middle image), IDH1 and 2, GCLC, SCO2 and ASS1 (in the right image). In the left and middle images, the respective lowest line represents the melting curve of the negative control, i.e. the curve for the sample with no cDNA in the qPCR reaction mixture. The graphs were generated using the software of the StepOnePlus™ Taqman machine (ABI, Applied Biosystems).

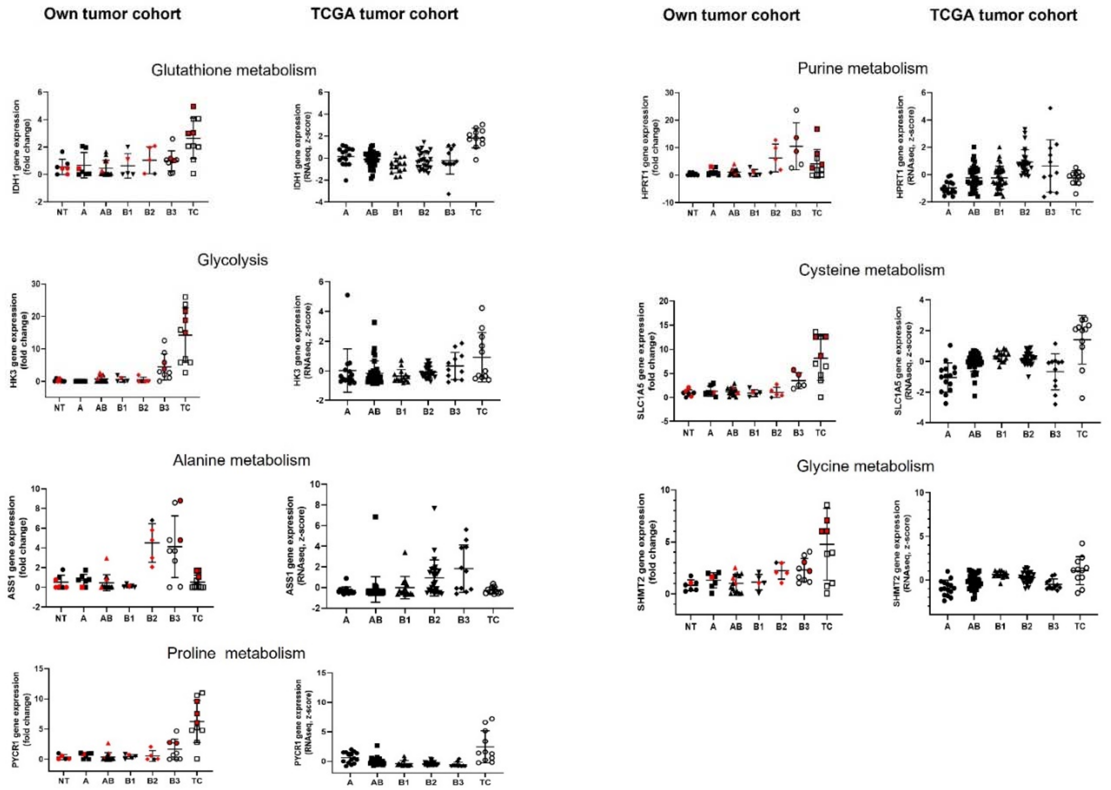

Figure S2. Relative gene expression levels (means ± SD) in our "Own tumor cohort" and the "TCGA tumor cohort. Seven representative genes were selected from each of the 7 metabolic pathways listed in Table 3. Relative gene expression levels (relative to GAPDH) were determined by qPCR in total RNA extracts from cryopreserved thymic tissue in our "Own tumor cohort". It comprised 51 thymic epithelial tumors (TETs) and 7 non-neoplastic thymi (NT), where the cohort of TETs consisted of 7 type A, 15 AB, 5 B1, 5 B2, 9 B3 and 10 thymic carcinomas (TC) and included the 15 cases studied by HRMAS-1H NMR (highlighted as red symbols). Relative gene expression values in our "Own tumor cohort" as depicted on the y-axis represent fold-changes relative to the gene expression in a normal thymus (case NT.1 from table 1). Gene expression values as extracted from The Cancer Genome

Atlas ("TCGA tumor cohort") database were based on RNA sequencing data and are depicted on the y-axis as z-scores [4]. The 115 cases of the "TCGA tumor cohort" for comparison consisted of 10 type A, 48 AB, 12 B1, 25 B2, 10 B3 thymomas and 10 TCs [4]. The two different analytical (qPCR versus RNAseq) and quantification methods (fold changes versus z-scores) preclude the direct comparison of the two cohorts. Nevertheless, the plots suggest that differences in relative expression of the 7 genes between TET types (e. g. between TCs and B3 thymomas) were largely concordant in our "Own" (Mannheim) and the "TCGA tumor cohort". Statistical analysis supported this impression (Table S6).

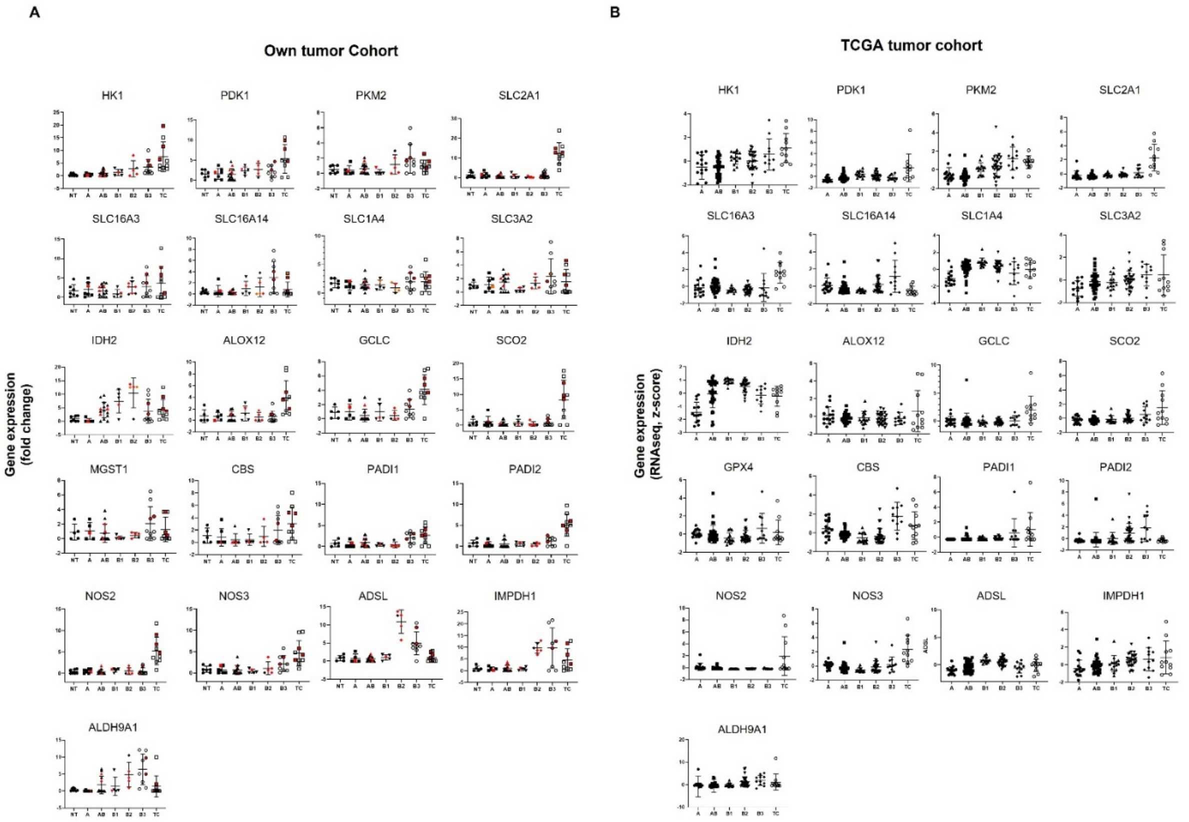

Figure S3. Relative gene expression levels (means ± SD) of 21 additional representative genes in our "Own tumor cohort" and the "TCGA tumor cohort". The additional genes (like those from Figure S2) were selected from each of the 7 metabolic pathways listed in Table 3. Relative gene expression levels in "Our tumor cohort" were determined by RT-qPCR in the same 51 thymic epithelial tumors (TETs) and 7 non-neoplastic thymi (NT) as described in Figure S2. The 15 cases among the 51 TETs studied by HRMAS-1H NMR are highlighted as red symbols. Relative gene expression values in "Our tumor cohort" as depicted on the y-axis represent fold-changes relative to the gene expression in a normal thymus (case NT.1 from table 1). Gene expression values as extracted from The Cancer Genome Atlas ("TCGA tumor cohort") database are depicted on the y-axis as z-scores that are derived from RNAseq data [4]. The 115 cases of the "TCGA tumor cohort" consisted of 10 type A, 48 AB, 12 B1, 25 B2, 10 type B3 and 10 TCs [4]. The two different analytical (qPCR versus RNAseq) and quantification methods (fold changes versus z-scores) preclude the direct comparison of the two cohorts. Nevertheless, the plots suggest that differences in relative expression of the 21 genes between TET types (e. g. between TCs and B3 thymomas) were largely concordant in our "Own tumor cohort" and the "TCGA tumor cohort". Statistical analysis supported this impression (Table S6).

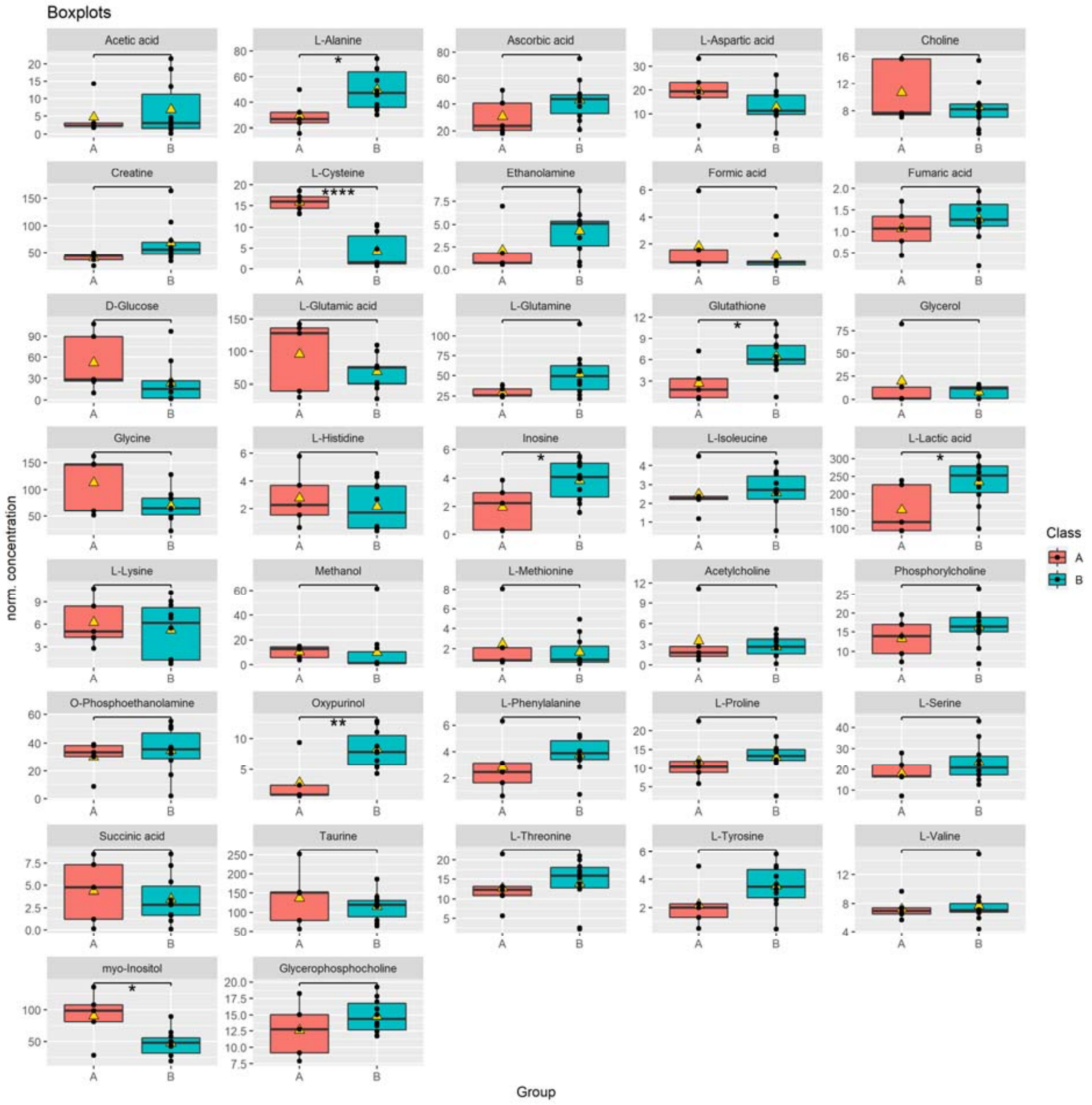

Figure S4. Concentrations of metabolites in the groups of clinically indolent (A) and aggressive (B) thymic epithelial tumors (TETs). Boxplots of the concentrations of metabolites in groups of indolent thymomas (type A, AB, B1 thymomas, red; $n = 5$) and aggressive TETs (type B2 and B3 thymomas and TCs, turquoise; $n = 10$). Data processing based on our raw data in Table S4 was performed by removing NT samples and performing zero imputing, normalization to the total sum of each sample and multiplying by 1000. The black bar shows the median of a distribution, while the yellow triangle shows the average. Each box is drawn from the 25 to the 75 percentiles. P-values of metabolites were determined with a Welch two sample t-test (see Table S3 for details). The seven metabolites with significantly different expression between the two groups are indicated by stars (* to ****) that represent p-values below 0.05, 0.01, 0.001 and 0.0001, respectively).

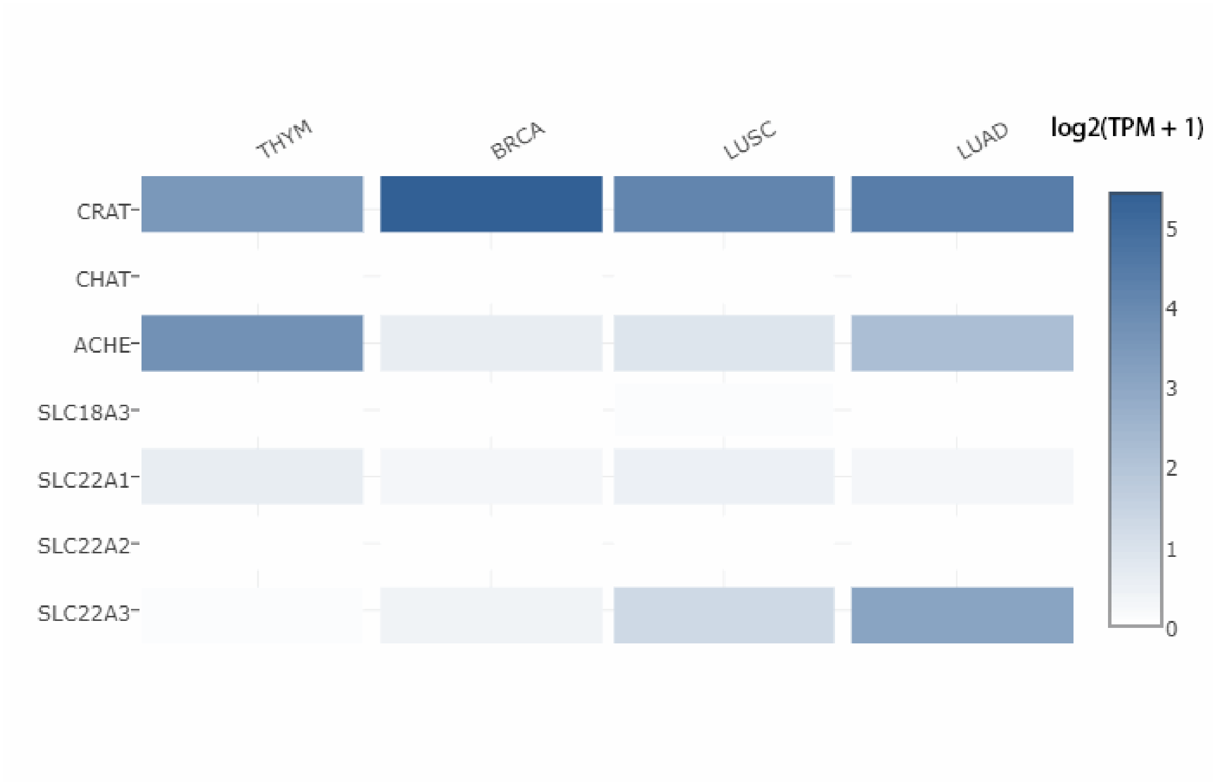

Figure S5. Comparative gene expression profiles (RNA) of genes related to acetylcholine (ACH) metabolism across the TCGA datasets of thymic epithelial tumors (THYM), breast cancers (BRCA) as well as lung squamous (LUSC) and lung adenocarcinomas (LUAD) (CBioPortal). CRAT (carnithine acetyltransferase) and CHAT (choline acetyltransferase) are involved in ACH synthesis; ACHE (acetylcholine esteras) is involved in ACH degradation; SLC18A3, SLC22A1, SLC22A2 and SLC22A3 are ACH transporters. The expression profile of TETs does not explain why ACH levels are higher in TETs than breast cancers (in which the ACH synthesizing CRAT is higher and ACH degrading ACHE is lower than in TETs). The heatmap is based on TCGA expression data and was generated using the GEPIA2 web server (Tang Z et al. Nucleic Acid Res 2019 Jul 2;47(W1):W556-560. doi: 10.1093(nar/ gkz430). We used log2 (TPM + 1) for the generation of the log-scale. TPM, Transcript Count Per Million.
