# Supplementary material for: Metabolic Profiling of Thymic Epithelial Tumors Hints to a Strong Warburg Effect, Glutaminolysis and Precarious Redox Homeostasis as Potential Therapeutic Targets
Source: Cancers (Basel). 2022 Mar 18;14(6):1564. doi: 10.3390/cancers14061564 (PMC8945961; doi:10.3390/cancers14061564)
Supplement: Supplementary file 1 [file cancers-14-01564-s001.zip › Supplementary Tables S1-S7.pdf]

Supplementary Tables

Table S1. Primers for quantifying mRNA levels of 28 selected genes in the 51 snap-frozen thymic epithelial tumors including the 15 samples, that were studied by HRMAS ^{1}H-NMR. GAPDH was used as housekeeping gene in the qPCR analysis and determination of relative gene expression levels using the ΔΔCt method. Gene names are given in Table S2.

Gene Name	Primer Forward	Primer reverse
ADSL	CCGCTCTTCCCTGGTCCAGTC	GATTTTCGTTTAATTCTCTTC
ALDH9A1	CCTGGAGCGAGTCCTCGGGTT	CCCGCTCCAGAACCTCGGCT
ALOX12	AGAAAAGTTGACTAGTCCAGTGT	AAAAGCTGTGCTAAACCAATTCCGA
ASS1	CTCCACGATGTCAATACGGC	CAACACCCCTGACATTCTCG
CBS	CCACATCACCACACTGCC	GCCGAACTTCTTCCCAATCT
GAPDH	TCGACAGTCAGCCGCATCT	CCGTTGACTCCGACCTTCA
GCLC	TGTCGCTGGGGAGTGATTTC	CAATGCCTTCCTGCAACAGC
HK1	AATGCTGGGAAACAAAGGT	AGAGGAATCCCTTCTTGGG
HK3	GTGGCATTCATGGAATGGG	AACCCTTGGTCCAGTTCAGG
HPRT1	TGGACAGGACTGAACGTCTTG	CCAGCAGGTCAGCAAAGAATTTA
IDH1	GGTAAAACCTATCATCATAGGTCG	CACATACAAGTTGGAAATTTCTGG
IDH2	GCTGCAGTGGGACCAC TATT	CTCTGCAGTACAAGGC CACA
IMPDH	TTCGTGCCCTACCTCATAGC	ATGGACCGAAGGACAGACAG
MGST1	ATTGGCCTCCTGTATTCCTTG	TAATCCCTCTGCTCCCCTCC
NOS2	GACTGTTGAGATGGAAGAAC	ATCTTCCTGTCTCCGAGGCG
NOS3	GTGATGGCGAAGCGAGTGAAG	CCGAGCCCGAACACACAGAAC
PADI1	CACAGCTCTGGTTGGCTTCA	CTGCACGTCCTTCAGCATCA
PADI2	TGAAGCACTCGGAACACGT	TTGTCACTGCTGGCCTCG
PDK1	CTGTGATACGGATCAGAAACCG	TCCACCAAACAATAAAGAGTGCT
PKM2	CCATTACCAGCGACCCCACAG	GGGCACGTGGGCGGTATCT
PYCR1	CCTACGGGGCTGCCAAGAT	ACTCT CCAGCACATGCAAGG
SCO2	TCACTCACTGCCCTGACATC	CGGTCAGACCCAACAGTCTT
SHMT2	CGAGTTGCGATGCTGTACTT	CTGCGTTGCTGTGCTGAG
SLC16A14	TGAAGACGACCGAAAGGCTAA	ATGTGAACAAAGAAGGACGAGAG
SLC16A3	CGTTCTGGGATGGGACTGAC	ATGTGCCTCTGGACCATGTG
SLC1A4	GACCGTACGGAGTCGAGAAG	GGGGGTTTCCTTCCTCAGTG
SLC1A5	AAGCACATCAGCCGTTTCAT	AGTGAGGACACCTCCAGCAG
SLC2A1	GGCCAAGAGTGTGCTAAAGAA	ACAGCGTTGATGCCAGACAG
SLC3A2	ACTTGGCTGAGTGGCAGAAT	AGATCGCTGGTGGATTCAAG

Table S2. Official full names of the 90 genes from the TCGA thymic epithelial tumor (TET) dataset [4] used for functional annotation clustering using 'DAVID'. The subset of 53 genes in bold represent the genes differentially expressed between individual TET types with similar lymphocyte contents but different (indolent versus aggressive) biological behavior (as detailed in Table S7). The subset of 28 genes whose expression was validated using qPCR in the Mannheim cohort of TETs are in red.

Gene abreviation	Official Gene Name
ACSL1	acyl-CoA synthetase long-chain family member 1
ADA	adenosine deaminase
**ADCY10**	**adenylate cyclase 10**
**ADSL**	**adenylosuccinase**
**ALDH4A1**	**aldehyde dehydrogenase 4 family member A1**
**ALDH9A1**	**aldehyde dehydrogenase 9 family member A1**
**ALDOA**	**aldolase, fructose-bisphosphate A**
**ALOX12**	**arachidonate 12-lipoxygenase, 12S type**
**ANPEP**	**Alanyl Aminopeptidase, Membrane**
**ARG1**	**arginase 1**
ARSD	arylsulfatase D
ASL	argininosuccinate lyase
**ASS1**	**argininosuccinate synthase 1**
BCAT1	branched chain amino acid transaminase 1
BCAT2	branched chain amino acid transaminase 2

CAD	carbamoylphosphate synthtase 2, aspartate transcarbamylase and dihydroorotase
**CBS**	**cystathionine-beta-synthase**
CNDP2	CNDP dipeptidase 2 (metallopeptidase M20 family)
DAO	D-amino acid oxidase
**DCK**	**deoxycytidine kinase**
DHCR24	24-dehydrocholesterol reductase
**FBP1**	**fructose-bisphosphatase 1**
**FOLH1**	**folate hydrolase 1**
**GART**	**phosphoribosylglycinamide formyltransferase, phosphoribosylglycinamide synthetase, phosphoribosylaminoimidazole synthetase**
**GCLC**	**glutamate-cysteine ligase catalytic subunit**
GFPT1	glutamine--fructose-6-phosphate transaminase 1
GFPT2	glutamine-fructose-6-phosphate transaminase 2
**GGT6**	**gamma-glutamyltransferase 6**
**GOT1**	**glutamic-oxaloacetic transaminase 1**
GOT2	glutamic-oxaloacetic transaminase 2
GPX1	glutathione peroxidase 1
GPX2	glutathione peroxidase 2
**GPX3**	**glutathione peroxidase 3**
**GPX4**	**glutathione peroxidase 4**
**GSTK1**	**glutathione S-transferase kappa 1**
**HK1**	**hexokinase 1**
HK2	hexokinase 2
**HK3**	**hexokinase 3**
**HPRT1**	**hypoxanthine phosphoribosyltransferase 1**
**IMPDH1**	**inosine monophosphate dehydrogenase 1**
**IDH1**	**isocitrate dehydrogenase (NADP(+)) 1, cytosolic**
**IDH2**	**isocitrate dehydrogenase (NADP(+)) 2, cytosolic**
KEAP1	kelch like ECH associated protein 1
**LAP3**	**leucine aminopeptidase 3**
MCCC2	methylcrotonoyl-CoA carboxylase 2
**MDH1**	**malate dehydrogenase 1**
**MGST1**	**microsomal glutathione S-transferase 1**
**MGST2**	**microsomal glutathione S-transferase 2**
NAT8L	N-acetyltransferase 8 like
NCF1	neutrophil cytosolic factor 1
NCF2	neutrophil cytosolic factor 2
**NIT2**	**nitrilase family member 2**
**NOS1**	**nitric oxide synthase 1**
**NOS2**	**nitric oxide synthase 2**
**NOS3**	**nitric oxide synthase 3**
**NT5C3A**	**5'-nucleotidase, cytosolic IIIA**
**NUDT2**	**nudix hydrolase 2**
**P4HA1**	**prolyl 4-hydroxylase subunit alpha 1**
**PADI1**	**peptidyl arginine deiminase 1**
**PADI2**	**peptidyl arginine deiminase 2**
PADI3	peptidyl arginine deiminase 3
PADI4	peptidyl arginine deiminase 4
PADI6	peptidyl arginine deiminase 6
PCK2	Phosphoenolpyruvate Carboxykinase 2
**PDK1**	**pyruvate dehydrogenase kinase 1**
PDK4	pyruvate dehydrogenase kinase 4
PFKFB3	6-phosphofructo-2-kinase/fructose-2,6-biphosphatase 3
PFKFB4	6-phosphofructo-2-kinase/fructose-2,6-biphosphatase 4
PFKM	phosphofructokinase, muscle
PHGDH	phosphoglycerate dehydrogenase
**PKM2**	**pyruvate kinase muscle 2**
**PNP**	**purine nucleoside phosphorylase**
**PRMT1**	**protein arginine methyltransferase 1**
**PRODH**	**proline dehydrogenase 1**
PSPH	phosphoserine phosphatase
**PYCR1**	**pyrroline-5-carboxylate reductase 1**
**RIMKLA**	**ribosomal modification protein rimK like family member A**

**SCO2**	**SCO2, cytochrome c oxidase assembly protein**
SDS	serine dehydratase
**SHMT2**	**serine hydroxymethyltransferase 2**
SLC16A1	solute carrier family 16 member 1
SLC16A2	solute carrier family 16 member 2
**SLC16A14**	**solute carrier family 16 member 14**
**SLC16A3**	**solute carrier family 16 member 3**
**SLC1A4**	**solute carrier family 1 member 4**
**SLC1A5**	**solute carrier family 1 member 5**
**SLC2A1**	**solute carrier family 2 member 1**
**SLC3A2**	**solute carrier family 3 member 2**
TAP1	transporter 1, ATP binding cassette subfamily B member (TAP1)
TAP2	transporter 2, ATP binding cassette subfamily B member (TAP2)

Table S3. Metabolites (n = 37) detected overall in 15 thymic epithelial tumors (TETs) using HRMAS ^{1}H-NMR spectroscopy. Given are p-values and incidences. Incidences represent the number of samples (of the 15 TETs investigated), in which the respective metabolite could be detected; in the other cases, metabolite concentrations were assumed to be below the detection threshold of the method and were set as zero in the raw data replaced by imputing during data processing for statistical evaluation as described in section 2.2. p-values concern the comparison of metabolite levels in the group of indolent TETs (type A, AB, B1 thymomas) and the group of aggressive TETs (type B2 and B3 thymomas and thymic carcinomas). Metabolites (n = 7) with significantly different levels between the two groups are shown in bold. p-values were determined by the Welch two sample t-test.

**Metabolite**	***p*-value**	**Incidence among 15 TETs**	**Metabolite**	***p*-value***	**Incidence among 15 TETs**
Acetic acid	0.593	13	**Inosine**	0.034	12
Acetylcholine	0.571	11	L-Isoleucine	0.926	11
Ascorbic acid	0.158	14	**L-Lactic acid**	0.049	15
**L-Alanine**	0.029	15	L-Lysine	0.597	9
L-Aspartic acid	0.174	11	**myo-Inositol**	0.015	15
Choline	0.318	15	Methanol	0.964	6
Creatine	0.138	15	L-Methionine	0.500	3
**L-Cysteine**	0.0000625	8	**Oxypurinol**	0.0094	10
Ethanolamine	0.157	8	Phosphorylcholine	0.312	14
Formic acid	0.444	2	Phosphoethanolamine	0.594	12
Fumaric acid	0.427	12	L-Phenylalanine	0.304	11
D-Glucose	0.169	10	L-Proline	0.721	12
L-Glutamic acid	0.210	14	L-Serine	0.294	13
L-Glutamine	0.095	14	Succinic acid	0.599	13
**Glutathione**	0.031	10	Taurine	0.452	15
Glycerol	0.334	8	L-Threonine	0.729	11
Glycine	0.056	15	L-Tyrosine	0.155	9
Glycerophosphocholine	0.235	15	L-Valine	0.719	14
L-Histidine	0.549	7			

Table S4. Metabolite concentrations in the 15 thymic epithelial tumors (TETs) and 4 non-neoplastic thymuses (NTs) as determined by HRMA 1H-NMR spectroscopy. Table S4 can be found in the excel file.

Table S5. "Area under the curve" (AUC) values obtained from receiver operating characteristic (ROC) curves analysis based on the metabolite levels determined in the 15 thymic epithelial tumors (TETs) analyzed in this study. Nine of the 37 metabolites, namely cysteine, alanine, glutathione, creatine, inosine, alanine, lactic acid, oxypurinol and myo-inositol showed an AUC value larger than 0.8. Of these nine metabolites, seven, i.e. all except creatine and glutamine, showed significantly different concentrations ($p < 0.05$) in indolent compared to aggressive TETs (Table S3).

**Metabolite**	**AUC**	**Metabolite**	**AUC**	**Metabolite**	**AUC**	**Metabolite**	**AUC**
Acetic acid	0.5	L-Glutamic acid	0.64	Methanol	0.74	L-Serine	0.66
Acetylcholine	0.54	L-Glutamine	0.82	L-Methionine	0.56	Succinic acid	0.56
Ascorbic acid	0.74	Glutathione	0.84	Oxypurinol	0.86	Taurine	0.6
L-Alanine	0.86	Glycerol	0.54	Phosphorylcholine	0.64	L-Threonine	0.62

L-Aspartic acid	0.7	Glycine	0.72	Phosphoethanolamine	0.56	L-Tyrosine	0.74
Choline	0.62	Glycerophosphocholine	0.66	L-Phenylalanine	0.72	L-Valine	0.56
Creatine	0.84	L-Histidine	0.62	L-Proline	0.7		
L-Cysteine	1	Inosine	0.82				
Ethanolamine	0.72	L-Isoleucine	0.58				
Formic acid	0.6	L-Lactic acid	0.82				
Fumaric acid	0.64	L-Lysine	0.58				
D-Glucose	0.76	myo-Inositol	0.84				

Table S6. *p*-values and significance levels calculated for the relative expression levels of the 28 genes from Figures S2 and S3 in three different comparisons: Aggressive thymic epithelial tumors (TETs, type B2 and B3 thymomas and thymic carcinomas (TC)) versus (vs) indolent TETs (type A, AB, B1 thymomas); TC vs all thymomas (A, AB, B1, B2, B3) and TC vs B3 thymomas in the Mannheim and TCGA cohorts. Two-tailed student's *t*-test was applied in the comparisons; ns: no significance. "Fairly similar" p-values (i.e. concordantly significant or non-significant differences between the two cohorts) are in black, discordant p-values are in red. In both cohorts, relative expressions showed "fair similarity" in 26 of 28 genes in the 1st comparison (aggressive vs indolent TETs), in 24 of 28 in the 2nd comparison (TC vs all thymomas) and in 21 of 28 in the 3rd comparison (TC vs type B3 thymomas).

Expressed Gene	B2,B3,TC vs A,AB,B1		TC vs A,AB,B1,B2, B3		TC vs B3	
	Mannheim	TCGA	Mannheim	TCGA	Mannheim	TCGA
ADSL	0.0001 ***	ns	ns	ns	0.0016 **	ns
ALDH9A1	0.007 **	0.0005 ***	ns	ns	0.0112 *	ns
ALOX12	0.0025 **	0.0.035 *	<0.0001 ****	0.0114 *	0.0044 **	ns
ASS1	0.0032 **	0.0002 ***	ns	ns	0.0025 **	0.006 **
PADI1	0.0046 **	0.0017 **	0.0004 ***	0.0003 ***	ns	ns
PADI2	0.0002 ***	0.0002 ***	<0.0001 ****	ns	0.0007 ***	0.0067 **
CBS	<0.0001 ****	0.0005 ***	ns	ns	ns	ns
GCLC	0.0011 **	0.047 *	<0.0001 ****	<0.0001 ****	0.0040 **	0.0161 *
HK1	0.0001 ***	<0.0001 ****	<0.0001 ****	<0.0001 ****	ns	ns
HK3	<0.0001 ****	0.016 *	<0.0001 ****	0.0432 *	0.0055 **	0.0374 *
HPRT1	<0.0001 ****	<0.0001 ****	ns	ns	ns	ns
IDH1	0.0002 ***	0.0025 **	<0.0001 ****	<0.0001 ****	0.0078 **	0.002 **
IDH2	ns	ns	ns	ns	ns	ns
IMPDH1	<0.0001 ****	0.0001 ***	ns	0.0347	ns	ns
MGST1	0.022 *	<0.0001 ****	ns	ns	ns	ns
NOS2	0.0022 **	0.00056 ***	<0.0001 ****	<0.0001 ****	0.0005 ***	0.0418 *
NOS3	0.0001 ***	0.0024 **	<0.0001 ****	<0.0001 ****	ns	0.006 **
PDK1	0.0038 **	<0.0001 ****	0.0003 ***	<0.0001 ****	0.030 *	0.027 *
PMK2	0.0015 **	<0.0001 ****	ns	0.0121*	ns	ns
PYCR1	<0.0001 ****	<0.0001 ****	<0.0001 ****	<0.0001 ****	0.0024 **	0.0017 **
SCO2	0.041 *	0.028 *	<0.0001 ****	<0.0001 ****	0.0026 **	ns
SHMT2	0.0001 ***	0.0112 *	<0.0001 ****	0.0002 ***	ns	0.0037 **
SLC16A14	0.0115 *	0.016 *	ns	ns	0.0462 *	0.0095 **
SLC16A3	0.035 *	0.0015 **	ns	<0.0001 ****	ns	0.01 *
SLC1A4	ns	ns	ns	ns	ns	ns
SLC1A5	0.003 **	0.0526 *	<0.0001 ****	<0.0001 ****	0.05 *	0.022 **
SLC2A1	0.0021 **	0.0001 ***	<0.0001 ****	<0.0001 ****	<0.0001 ****	0.0022 **
SLC3A2	ns	0.0003 ***	ns	ns	ns	ns

*<0.05; **<0.01; ***<0.001, ****<0.0001.

Table S7. Comparison of relative expression levels of 53 genes between indolent thymic epithelial tumors (TETs, i.e. type A, AB and B1 thymomas) and aggressive TETs (type B2 and B3 thymomas

and thymic carcinomas (TC) of the TCGA cohort taking the contents of lymphoid cells into account. Lymphoid cell contents were typically low in type A and B3 thymomas and TC but high in type AB, B1 and B2 thymomas (Table 1). Statistical analysis of comparisons of type A with B3 thymomas, type A thymomas with TC (lymphocyte-poor TETs) and of AB plus B1 thyomas with B2 thymomas (lymphocyte-rich TETs). Two-tailed student's *t*-test and one-Way ANOVA were applied in the A vs B3, type A vs TC and AB+B1 vs B2 comparisons, respectively.

Gene	A vs TC	A vs B3	AB+B1 vs B2
ADCY10	0.0002 ***	ns	0.0011 **
ADSL	0.0141 *	ns	<0.0001 ****
ALDH4A1	ns	ns	0.0009 ***
ALDH9A1	ns	0.038 *	0.0009 ***
ALDOA	0.0125 *	0.0165 *	<0.0001 ****
ALOX12	0.0037 **	ns	ns
ANPEP	ns	ns	<0.0001 ****
ARG1	0.0403 *	<0.0001 ****	0.0003 ***
ASS1	ns	0.0013 **	0.007 **
CBS	ns	0,0123 *	ns
DCK	ns	ns	0.0032 **
FBP1	0.0091 **	ns	0.0056 **
FOLH1	0.0091 **	ns	0.0059 **
GART	<0.0001 ****	0.0435 *	0.0013 **
GCLC	0.0053 **	ns	ns
GGT6	<0.0001 ****	ns	0.0099 **
GOT1	0.0418 *	0.0235 *	<0.0001 ****
GSTK1	ns	ns	0.0064 **
HK1	0.0043 **	0.0045 **	<0.0001 ****
HK3	ns	ns	0.0004 ***
HPRT1	0.0002 ***	0.0029 **	<0.0001 ****
IDH1	<0.0001 ****	ns	ns
IDH2	0.0006 ***	0.0002 ****	0.0003 ***
IMPDH1	0.0339 *	0.0272 *	0.0068 **
LAP3	<0.0001 ****	ns	ns
MDH1	ns	ns	<0.0001 ****
MGST1	0.0015 **	<0.0001 ****	0.0159 *
MGST2	0.043 *	0.0163 *	0.0008 ***
NIT2	0.0012 **	0.0018 **	0.0238 *
NOS2	0.0445 *	ns	0.0029 **
NOS3	0.0004 ***	ns	ns
NT5C3A	ns	ns	0.0134 *
NUDT2	ns	ns	0.0263 *
P4HA1	ns	ns	0.0363 *
PADI1	0.0311 *	ns	<0.0001 ****
PADI2	ns	0.0016 **	0.0089 **
PCK2	ns	ns	0.001 **
PDK1	0.0029 **	ns	<0.0001 ****
PKM2	0.0001 ***	0.0002 ***	<0.0001 ****
PNP	<0,0001 ****	0,0111 *	0.015 *
PRODH	ns	ns	0.0417 *
PSPH	ns	ns	0.0001 ***
PYCR1	0.0226 *	0.0005 ***	0.0006 ***

RIMKLA	0.0251 *	0.0062 **	<0.0001 ****
SCO2	0.0098 **	0,0296 *	ns
SDS	0.0002 ***	ns	0.0011 **
SHMT2	0.0005 ***	ns	0.0006 ***
SLC16A14	0,0378 *	ns	0,012 *
SLC16A3	0.0004 ***	ns	0.0008 ***
SLC1A4	0.0117 *	ns	0.006 **
SLC1A5	<0.0001 ****	ns	ns
SLC2A1	<0.0001 ****	ns	0.002 **
SLC3A2	0.0379 *	ns	ns

*<0.05; **<0.01; ***<0.001, ****<0.0001.
